# Modern Trends for Peripheral Nerve Repair and Regeneration: Beyond the Hollow Nerve Guidance Conduit

**DOI:** 10.3389/fbioe.2019.00337

**Published:** 2019-11-22

**Authors:** Cristiana R. Carvalho, Joaquim M. Oliveira, Rui L. Reis

**Affiliations:** ^1^3B's Research Group, I3Bs – Research Institute on Biomaterials, Biodegradables and Biomimetics, University of Minho, Headquarters of the European Institute of Excellence on Tissue Engineering and Regenerative Medicine, Guimarães, Portugal; ^2^ICVS/3B's - PT Government Associate Laboratory, Braga/Guimarães, Portugal; ^3^The Discoveries Centre for Regenerative and Precision Medicine, Headquarters at University of Minho, Avepark, Guimarães, Portugal

**Keywords:** peripheral nerve, tissue engineering, biomaterials, nerve guidance conduit, luminal fillers

## Abstract

Peripheral nerve repair and regeneration remains among the greatest challenges in tissue engineering and regenerative medicine. Even though peripheral nerve injuries (PNIs) are capable of some degree of regeneration, frail recovery is seen even when the best microsurgical technique is applied. PNIs are known to be very incapacitating for the patient, due to the deprivation of motor and sensory abilities. Since there is no optimal solution for tackling this problem up to this day, the evolution in the field is constant, with innovative designs of advanced nerve guidance conduits (NGCs) being reported every day. As a basic concept, a NGC should act as a physical barrier from the external environment, concomitantly acting as physical guidance for the regenerative axons across the gap lesion. NGCs should also be able to retain the naturally released nerve growth factors secreted by the damaged nerve stumps, as well as reducing the invasion of scar tissue-forming fibroblasts to the injury site. Based on the neurobiological knowledge related to the events that succeed after a nerve injury, neuronal subsistence is subjected to the existence of an ideal environment of growth factors, hormones, cytokines, and extracellular matrix (ECM) factors. Therefore, it is known that multifunctional NGCs fabricated through combinatorial approaches are needed to improve the functional and clinical outcomes after PNIs. The present work overviews the current reports dealing with the several features that can be used to improve peripheral nerve regeneration (PNR), ranging from the simple use of hollow NGCs to tissue engineered intraluminal fillers, or to even more advanced strategies, comprising the molecular and gene therapies as well as cell-based therapies.

## Introduction

Insights of neuronal injury and repair date back to early periods, specifically to Galen in the second century AD (Nawabi et al., 2006). The research on this topic has been rising continuously and several nerve repair techniques have progressed with time. Despite this fact, the status of peripheral nerve injuries (PNIs) and peripheral nerve regeneration (PNR) has always been in the shadow of the neurosurgery field. It is regarded has less significant when compared to areas such as central nervous system (CNS) disorders, which are seen as more prominent, tougher and therefore perceived as a more distinguished and notable field. In fact, it has been estimated that ~2–3% of all patients admitted to a Level I trauma centers suffer from PNIs (Noble et al., 1998) while cervical spine injury occurs in up to 3–6% of all patients with trauma (Ghafoor et al., 2005). This means that the CNS injuries are almost doubled when compared to the peripheral ones, which also carries higher costs. The main reason appointed to this is based on the word “peripheral” itself, as it suggests lesser relevance and difficulty. Furthermore, to increase this fallacy, several forged ideas increase the devaluation of this field, such as the idea that PNIs are irreversible, that peripheral nerves do not have any capacity to regenerate, and the results of peripheral nerve surgery are insignificant to the patient (Rasulić, 2018). However, although peripheral nerve repair is not a life-saving surgery, it has been proved that it is a life-changing surgery, with significant benefits in the patient's quality of life. Also, since most patients with PNIs fit in the working-age population, peripheral nerve repair also has substantial socioeconomic implications (Wojtkiewicz et al., 2015). After decades of investigation, it is becoming progressively clear that peripheral nerve repair is not a “peripheral” area and the full attention by the part of clinicians and scientists is needed to overcome this public-health concern.

Peripheral nerves provide the path for all types of axons that compose the peripheral nervous system (PNS), (e.g., motor (afferent), sensory (efferent) axons). Injuries to these nerves are common conditions, due to their scarce physical protection (unlike the CNS, which is protected by bone and layers of meninges) and superficial position throughout the human body. Depending on the injury, an extensive array of symptoms and outcomes are possible. They will be contingent on the severity, type of trauma, age, and type of nerves involved (Siemionow and Brzezicki, 2009). Although much awareness and information already exist on the natural mechanisms of injury and regeneration, effective regenerative treatments that ensure complete functional and sensory recovery are rare (Grinsell and Keating, 2014; He et al., 2014).

**Figure 1:**
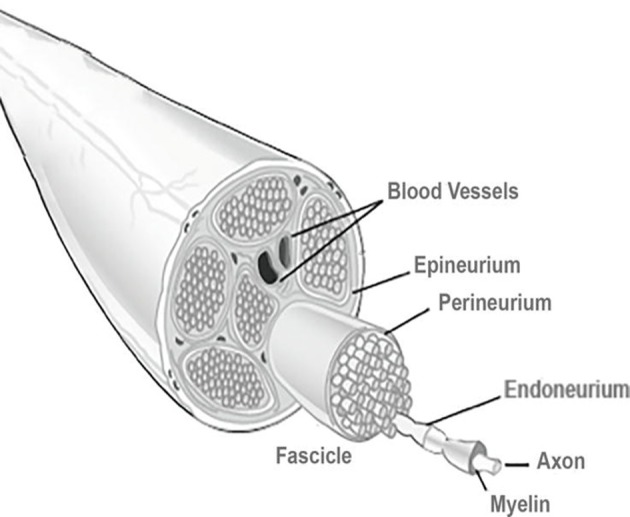
Basic anatomy of a peripheral nerve. A connective tissue layer, endoneurium, involves the individual axons. An arrangement of axons, designed fascicles, is surrounded by the perineurium, and groups of fascicles are separated by the epineurium. External to this layer is the blood supply derived from major arteries and the latter is involved by the mesoneurium. Reproduced with permission from Pedrosa et al. (2016).

**Figure 2:**
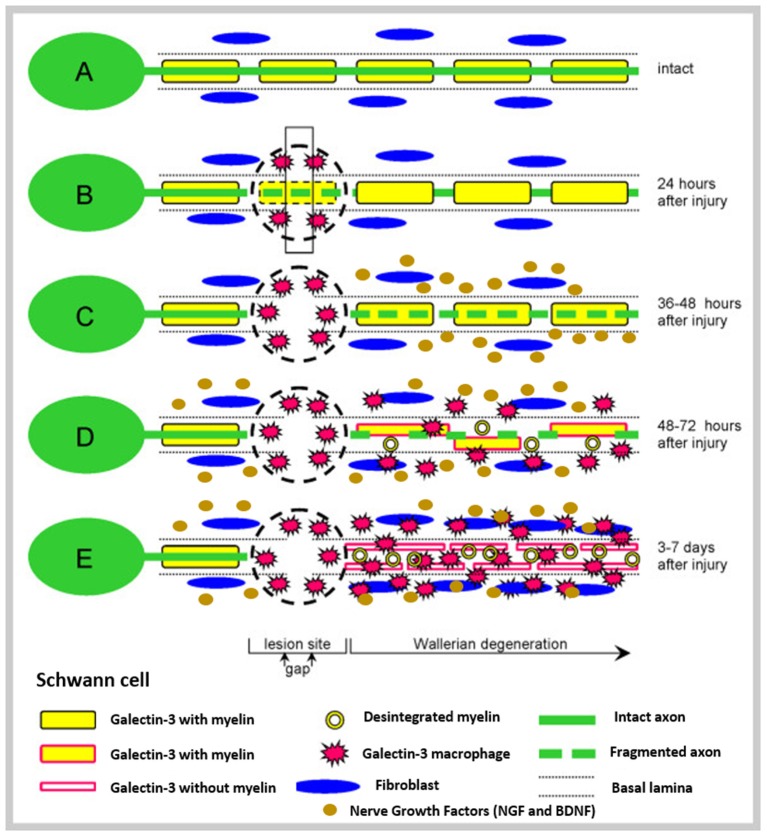
Schematic representation of injury and regenerative process involved in peripheral nerves. **(A)** Represents the intact nerve, with myelin enwrapping healthy axons; **(B)** The moment where an injury occurs, instantaneous tissue damage happens at the injury site. After a few hours, macrophages gather at the lesion; **(C)** The normal Wallerian degeneration process starts roughly 1 day after the initial trauma and axons start to disintegrate; Growth factors are released by Schwann cells in the distal end. **(D)** Enrolment of Galectin-3 macrophages, which contribute to myelin degradation and removal of myelin debris. Growth factors are retrogradely transported to the proximal end, toward the cell body; and **(E)** The typical degradation of the distal nerve end happens with the participation of the Galectin-3 macrophages and Schwann cells. These cellular components scavenge deteriorated myelin and axonal matter. After the clearance of the debris, Schwann cells proliferate and align, forming the Bunger bands, for future guiding of the regenerating axons. Reproduced with the permission from Rotshenker (2011).

To deep understand the phenomena of nerve injury and repair, the basic anatomy of peripheral nerves must be known (Figure 1). After the injury, the process of Wallerian degeneration starts immediately (Rotshenker, 2011). In brief, nerve stumps distal to the injury site will experience cellular variations despite the fact that the cells themselves were not injured in the first place. Axons starts to collapse, Schwann cells discard their ensheathing myelin and macrophages are recruited. The later are employed to remove degenerated axons and myelin debris, along with Schwann cells (Deumens et al., 2010). Furthermore, after a few days, Schwann cells de-differentiate owing to their lost connection with axons, starting a vigorous proliferation. Galectin-3 is known to play a key role in activating myelin phagocytosis. In this process, macrophages and Schwann cells are promoted to degrade myelin, thus having a major importance in the degeneration process (Pesheva et al., 2002). Both types of Schwann cells, the pre-existent and the recently produced Schwann cells, align together to form the bands of Bungner, which are highly aligned fibers formed by the basal lamina of the Schwan cells. These bands are key topographical cues responsible for guiding the axon and their growth cones, from the proximal to the distal site, across the gap. In optimal conditions, the growth cones can extend at a rate of 1–3 mm a day (Griffin et al., 2013). Overall, Schwann cells affect PNR in three distinct manners: (i) proliferation, (ii) development of bands of Bungner, and (iii) secretion of adequate growth factors (GF) (Jessen et al., 2015). Figure 2 depicts the process of injury and regeneration of peripheral nerves.

Herein, we aim to summarize the necessary concepts to fully understand the phenomena of PNIs and regeneration, which pose complex and demanding challenges in tissue engineering and regenerative medicine (TERM).

## Tissue Engineering and Regenerative Medicine Concepts for PNR

Tissue engineering (TE) is a vital instrument in the field of regenerative medicine (RM), which has been the subject of dynamic scientific research in the last decades (Langer and Vacanti, 1993). The two terms have been referred to as TERM altogether (Furth et al., 2007). TERM tactics exist in a variety of human tissues and organs. The pivotal point is that these strategies bring new therapeutic possibilities, not only to general population, but more specifically to young patients and professional athletes and sportsman, allowing their reintegration and rebuilding of biological functions.

Overall, TERM strategies assist in the re-establishment, support, regeneration, or replacement of injured tissues and organs (Furth et al., 2007). Traumatic offense, oncological resection, congenital malformations, or progressively degenerative diseases result on tissues and organs that need replacement.

**Figure 3:**
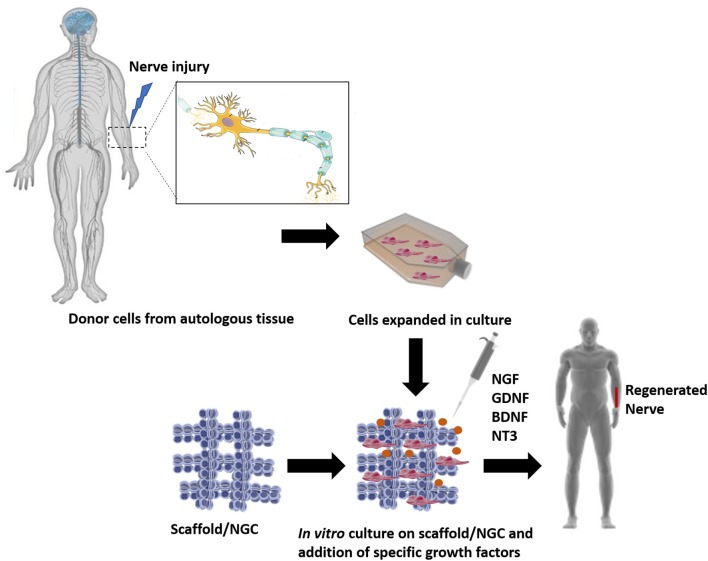
The classic TE model, where a triad of components interact with each other: scaffolds, cells and biological molecules. Overall, it includes the combination of living cells isolated from the patient donor tissue (nerve) and expanded in culture, with a natural, synthetic, or bioartificial matrix or scaffold. Together with the addition of biological stimuli such as growth factors, a 3D living construct that is structurally, mechanically, and functionally equal to a nerve tissue. The engineered construct can be implanted in the patient in order to restore the damaged tissue.

TERM strategies propose to use the ideologies of several fields, such as cell relocation, material science, nanotechnology and bioengineering to fabricate biological replacements of the damaged organs, eliminating the need and the wait of a transplant. Since its start, TE and now TERM, have been relying on three pillars: (i) scaffolds, (ii) cells, and (iii) growth factors (GF) (Langer and Vacanti, 1993). The basic strategies used in TERM approaches, applied to PNR, can be seen in Figure 3.

To achieve the ambitioned regeneration, several strategies can be used. One of them is the use of acellular matrices, composed only of smart biomaterials, which can react to the fluctuations in the environment when one of its property changes by the exterior conditions. They can either be prepared by manufacturing artificial scaffolds or by preparing acellular materials after decellularization of tissues (Moore et al., 2011). Cellular components can also be added and advanced medicinal therapeutic products (ATMPS) are obtained (Hu et al., 2016). For instance, Hu et al. (2016) used a 3D-printing technology to manufacture a bio-conduit. It consisted of a cryo-polymerized gelatin gel to which was further added adipose-derived stem cells (ADSCs). In a different approach, Dai et al. co-cultured Schwann cells and dental pulp stem cells on poly(d,l-lactide) (PLA) NGCs and assessed their effectiveness for restoring a 15 mm long critical gap defect in the rat sciatic nerve.

In both strategies, acellular or cellular materials, the biomaterial transformed in a scaffold must provide mechanical sustenance and proper features that contribute to tissue regeneration and formation, as the seeded cellular components cells proliferate and acquire the right morphology (Atala, 2004; Furth et al., 2007). Furthermore, nerves are exposed to several types of stress placed upon them, such as tensile, shear and compressive forces, which arise from postures or movements. Therefore, the scaffolds used in attempt to regenerate nerves must withstand such forces (Topp and Boyd, 2006). Materials used in this scope must have mechanical resistance to hold the regenerating nerve, however matching the mechanical and physical properties of the native nerve. Allied to that, properties such as tensile strength, suturability, and appropriate swelling and degradation must meet the required necessities (Nectow et al., 2012).

Another TERM strategy focuses on the use of cells alone, without the use of biomaterials, in which cells organize by self-organization and self-assembly (Shimomura et al., 2018). Altogether, regardless of the strategy followed, TERM aims at the creation of regenerated tissue for the rebuilding of the damaged parts in the body, resulting in substantial therapeutic benefits for patients for whom there are not currently any clinically effective therapies.

An immense body of research has been published regarding PNI and PNR (Mobini et al., 2017; Tajdaran et al., 2019; Tomita et al., 2019). However, regrettably, TERM strategies are not yet delivering significant progress in terms of clinical outcomes and commercialization, as there has not been a substantial translation from the bench to the clinics. This applies specifically to the case of PNR. Despite intensive research and plentiful approaches that have been published, none have achieved the desired results.

### The First Concept: Regeneration Within a Hollow Conduit

The application of hollow conduits for nerve repair was first projected in 1881, in which a hollow bone was used to bridge the nerve gap in a dog model, nevertheless with poor results (IJpma et al., 2008). The hollow tubulization technique aims to isolate the re-developing axons from fibrotic tissue, protect the regenerating nerve from inadequate mechanical forces, longitudinally guide the new-forming tissue and condense the growth factors secreted by Schwann cells between the nerve stumps (Lundborg et al., 1982). Nowadays, we possess extensive knowledge about Wallerian degeneration and the natural mechanisms of repair (Rotshenker, 2011). Taking this information in consideration, all the evidence points to the advantages related to the addition of luminal fillers to NGCs. Despite that fact, the extensive usage of hollow NGCs remains the common practice in clinical settings, due to their extensive FDA-approval.

**Figure 4:**
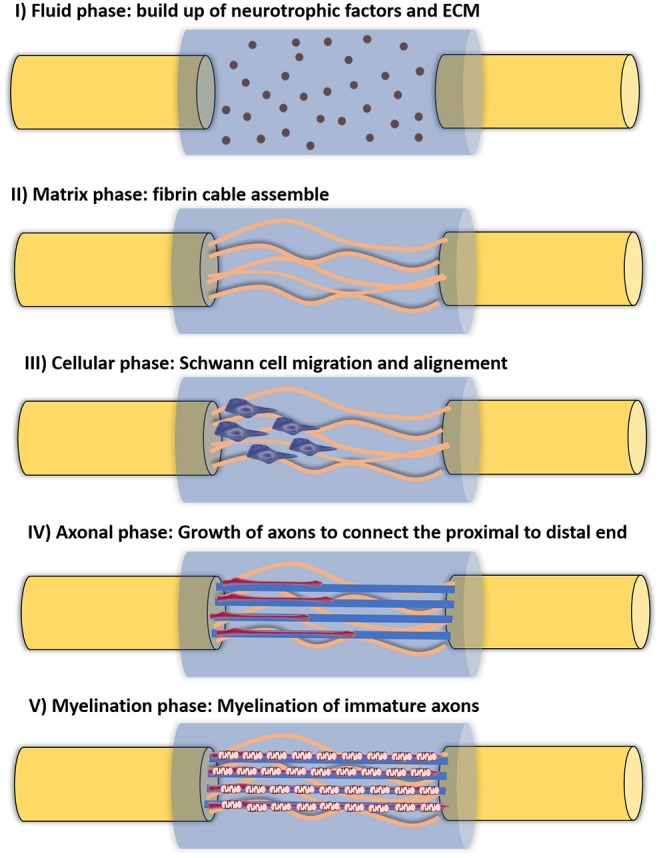
Five different phases of nerve regeneration inside a hollow NGC. The phase corresponds to the sequenced phases of Wallerian degeneration and resulting regeneration mechanism. Phase I corresponds to the fluid phase, where the conduit is filled with plasma exudate containing neurotrophic factors and ECM molecules. This phase takes place a few hours after injury. Phase II corresponds to the matrix formation, where fibrin cables are formed along the gap around 1 week after injury. Phase III is the cellular phase, where Schwann cells invade the gap, migrate and proliferate. They tend to align along the fibrin cable, forming the Bands of Bungner. Phase IV is axonal phase, which occurs around 2 weeks after injury. The re-growing immature axons use the biological cues provided by Schwann cells to reach their distal targets. Phase V corresponds to the myelin phase. At this time, around 3 weeks post-injury, Schwann cells shift to a myelinating phenotype and produce myelin which is wrapped around each axon, forming the mature myelinated axons.

The use of hollow NGCs has already shown to be advantageous in some situations, as they can prevent the permeation of fibroblasts and enable the buildup of neurotrophic factors (NTFs), which lead to regenerating cues. Furthermore, it has been proved that hollow conduits prevent neuroma and scar formation, ineffective axon sprouting and fibroblast intrusion. Taking advantage of the initially used impermeable silicon conduits, Williams et al. (1983) described the different stages of regeneration inside a hollow NGC, in a 10 mm rat sciatic nerve gap. It follows five different phases and can be seen in Figure 4. Briefly, the first phase is the fluid phase, where the conduit is filled with fluid containing NTFs, around 12 h after the injury. Within the first week, the second phase arises (the matrix phase), where the fibrin cable is formed. It follows the third phase, where the cellular migration starts to occur. Here, the cellular components migrate through the fibrin matrix. The axonal phase is phase four, where the first axons are visible migrating from the proximal nerve end, as early as 2 weeks after the injury. The final phase is the myelination phase, occurring around week 4 after injury (Shoichet and Midha, 2004). However, it is known that hollow NGCs can contribute to incomplete reinnervation, owing to axon dispersal and/or poly-innervation of diverse target tissues by axonal fibers belonging to same neuron. Therefore, hollow lumen NGCs are suggested for small gaps (up to 10 mm lesions) in the sensory nerves (Du et al., 2018).

Hollow cylindrical tubes can be fabricated by several techniques, such as electrospinning, crosslinking, physical film rolling, injection molding, melting extrusion and braiding (Sarker et al., 2018). In the previous decades, numerous NGCs made of both synthetic and natural biomaterials have been reported.

Nevertheless, there have been advances on this specific issue. In 2014, the FDA-cleared the first NGC comprising a 3D luminal filler, named NeuraGen 3D Nerve Guide Matrix® The NGC itself is made of collagen I and the luminal filler comprises a blending of collagen I and glycosaminoglycan chondroitin-6-sulfate. Furthermore, topographical cues are present in the luminal filler, as aligned porosity may act as a cue for new and expanding axons. The benefit of such technology was proved *in vivo*, where there as a visible improvement in nerve regeneration as compared to the counterpart hollow NGC (Lee et al., 2012).

However, one must keep in mind that although hollow conduits do not fully solve the obstacles faced in the clinics, they are subject to a wider clinical acceptance when compared to more complex types of conduits, associated to a *in vivo* better performance For instance, it has been proved that adding the 3D luminal filler to the NeuraGen conduit increased its potential of regeneration to limits similar to the autograft (Lee et al., 2012). In another study, by adding PLGA microspheres capable of releasing GDNF embedded in a fibrin gel to the lumen of the conduit (Tajdaran et al., 2016), it increased the numbers of regenerating motor and sensory neurons to levels similar from those observed with immediate nerve repair.

### Intraluminal Guidance Structures

A dual criterion is relevant for the success of a NGCs: the type of biomaterial used and its architectural features (Mukhatyar et al., 2011). Succinctly, in longer gaps, the formation of the fibrin cable is compromised. Therefore, Schwann cells are incapable of aligning through the injury site, diminishing the formation of the Bands of Bungner, the indispensable topographical guidance structures for re-growing axons (Hoffman-Kim et al., 2010). Hence, to control the scattering of the axons inside the hollow conduits, many approaches focus on filling the lumen with a diversity of shapes, with the objective of being biomimetic and looked at as alternatives to autografts.

In one hand, an unfilled lumen carries the benefit of permitting enough space for free nerve regeneration, in which the axons are permitted to re-innervate their suitable target. On the other hand, a lumen which is occupied with any type of luminal can provide a supporting structure, either mechanical or biological, that favors cells ingrowths, guidance, and correct targeting (Meyer et al., 2016b; López-Cebral et al., 2017). In fact, proliferating axons grow distal tip expansions named growth cones, which function is to discover and identify proper cues within the surrounding environment, through their filopodia and lamellipodia, at a nanoscale range (Lundborg, 2000). Thus, the architecture of the NGC's interior is expected to be a crucial factor in order to achieve an effective axon growth across the gap. This may be the reason why single hollow NGCs are limited to 10 mm nerve gaps. Topographical cues may alter cell shape and act together with biochemical environmental cues. However, the mechanism of cellular response to topographical cues is yet to be fully elucidated (Thomson et al., 2017).

**Figure 5:**
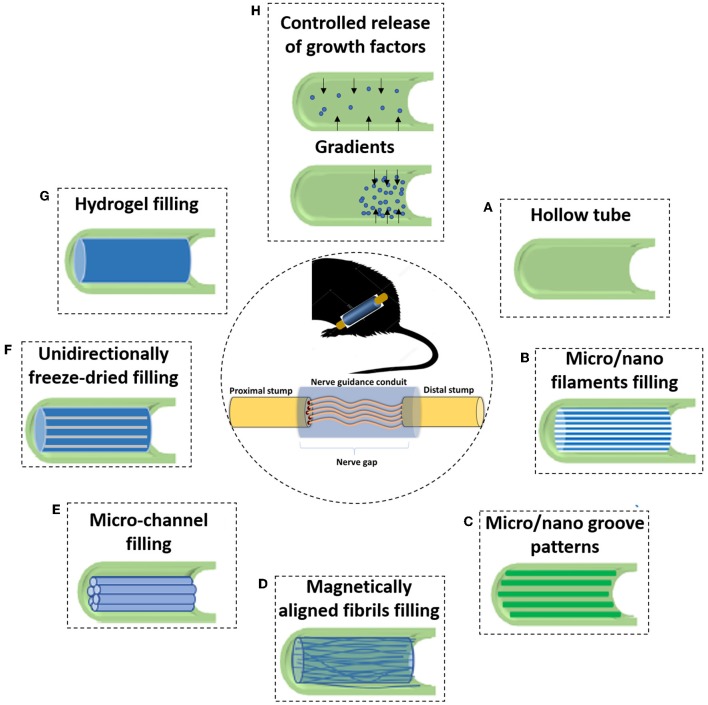
Due to the incapacity of hollow NGCs to bridge larger nerve gaps, various filler materials and designs have been developed to enhance the performance of NGCs. **(A)** The initial strategy consisted of simple hollow NGCs; When considering luminal fillers, experiments suggested that the regenerating axons prefer aligned features rather than random orientation. Therefore, many of the approaches focus on obtaining an anisotropic topography. With this strategy in mind, many types of luminal matrices are considered **(B)** Micro- or nano- filaments resembling the structure of endoneurial tubes; **(C)** Micro/nano groove-patterns; **(D)** Magnetically aligned fibrils or cells; **(E)** Micro-channel filling; **(F)** Unidirectionally freeze-dried; **(G)** Another strategy consist in inserting permissive hydrogels as luminal fillers, being a soft support to regenerative axons; and **(H)** One branch of PNR research also focuses on the controlled delivery of growth factors. That can be achieved, for instance, using crescent gradients of growth factors from the proximal to the distal sites, acting as a biochemical cue and attracting regenerating neurons to reach the final target.

A variety of strategies have been explored by scientists in order to attempt to substitute the sustenance and directionan cues provided by the natural ECM tissue cable. NGCs have been filled with all kinds of materials (Chen et al., 2006), such as hydrogels (Guo et al., 2017), nanofibers (Zor et al., 2014) or membranes (Meyer et al., 2016a), among others. Some of the most relevant adopted strategies to go beyond a hollow NGC can be seen in the depicted scheme in Figure 5. Furthermore, traditional and rapid prototyping techniques have been used to prepare the luminal fillers for NGCs. The classic systems used in the field of nerve regeneration comprise electrospinning (Belanger et al., 2018), poro-leaching (Knight and Przyborski, 2015), freeze-drying (Carvalho et al., 2018a), and solvent- or thermally- induced phase separation (Liu et al., 2015). Instead, rapid prototyping techniques which are critically controlled and software-driven, allow the meticulous layer-by-layer manufacture of scaffolds which have been on the rise due to their outstanding capacities, namely the 3D printing and bioprinting (Petcu et al., 2018).

The anisotropic architecture of the substratum has also demonstrated to be an important feature of native nerve tissue and in the process of Wallerian degeneration. An extensive number of reports advocate that topographical guiding cues are, similar to those delivered by the natural basal lamina aligned structures, indispensable to position axonal growth in the nerve gap (Perretta and Green, 2017). In fact, since 1934 that it has been exposed that the aligned topography of clotted blood plasma can serve as paths for guiding the DRG's axon elongation (Noble et al., 1998). Aligned structures, with parallel linear grooves, pores, or fibers have been shown to promote neurite outgrowth in PNR (Bellamkonda, 2006). This alignment induces cell migration and organization along the nerve on a preferred direction, from the proximal to the distal stump (Huang Y.-A. et al., 2018). With intensive research in this field, it is now known that aligned topographical cues enhanced neurite outgrowth by activating mTOR pathway (Thomson et al., 2017). From the molecular point of view, mTOR gene expression is at its peak between 48 and 72 h after positioning of the cells, which coincides with the commencement of rapid and aligned neurite outgrowth. Thus, this study showed that mTOR levels significantly increased by patterned topography.

#### Hydrogel Fillings

Considering hydrogel fillings, a variety of molecules such as specific proteins, ECM, polysaccharides, and peptides have been applied to formulate hydrogel matrices to be placed in the lumen of hollow NGCs. However, reports on the successful nerve regeneration through the use of hydrogels have been contradictory. For instance, self-assembling peptide RADA16-Mix in the form a 3D hydrogel presented to be a suitable environment for sciatic nerve regeneration, when functionalized with IKVAV and RGDs. This hydrogel was injected inside an electrospun PLLA NGC (Wu et al., 2017). In this specific study, when implanted in a rat, the nerves migrated right through the RADA 16-Mix hydrogel toward the distal end. Furthermore, the same formulation encouraged more axonal regeneration and Schwann cells migration. This leads to higher functional recovery detected by the gait-stance duration percentage and the formation of new neuromuscular junction structures. In another study where peptides were combined with hydrogels (McGrath et al., 2010), a tubular conduit was filled with synthetic matrix BD™ PuraMatrix™ peptide hydrogel and seeded with Schwann cells. The peptide matrix self-assembles to form a fibrous 3D hydrogel structure due to the presence of certain amino-acids, under physiological conditions. This device was tested *in vivo* on a 10 mm sciatic nerve defect in adult rats. After extensive characterization and several timepoints analyzed, it was found that the presence of Schwann cells in the BD hydrogel expressively augmented the capacity for axonal elongation in short-term experiments. However, it fails to endure long-term neuronal regeneration and prevent muscle atrophy. Du et al. (2017) developed a 3D fibrin hydrogel that proved to be a suitable microenvironment by imitating the native fibrin cable inside the NGC. In this study, the developed fibrin hydrogel with hierarchically aligned topography presented low elasticity (~1.5 kPa) that is similar to nerve ECM and to the native fibrin cable. After an *in vivo* study in a 10 mm defect model in rat, it was established that the hydrogel provided a positive environmental setting to provision Schwann cell cable formation and quicken axonal regeneration with better-quality motor functional recovery.

On the other hand, NVR-Gel (Meyer et al., 2016b) or Gellan Gum (Carvalho et al., 2018b) placed inside chitosan NGC obviously impaired axonal outgrowth. In the first study, Cora et al. reconstructed a critical length nerve defect (15 mm) with chitosan NGCs filled with NVR-Gel. While autologous nerve grafts provided functional sensory and motor regeneration in all of the animals (100%), the presence of NVR-Gel into the chitosan nerve guides visibly reduced axonal regeneration, physically blocking the regenerated nerves. The same happened to Carvalho et al. (2018b). In this study, different Gellan Gum formulations were injected inside the same chitosan conduits in a 10 mm sciatic nerve defect in rats. After 3, 6, and 12 weeks, functional and histomorphological *in vivo* assays showed that it did not lead to enhanced nerve regeneration, comparing with hollow nerve guidance channels. That was due to excessive density of the filler material.

Therefore, it can be concluded that phenomena such as inappropriate positioning, erratic distribution or excessive density of intraluminal hydrogel fillers might result in regeneration deficiency, at least at short time of implantation. Therefore, *in vivo* studies to assess the efficiency of hydrogels as lumen fillers of NGCs functionality at long term (e.g., 6–12 months) are needed, in future.

#### Freeze-Dried Isotropic Cues

Topographical cues are influential controllers of the amount and orientation of neurite elongation (Thomson et al., 2017). One way to achieve an anisotropic 3D matrix is with controlled freeze-dried technique. More specifically, freeze-casting is a manufacturing method that allows the production of porous matrices containing a controlled and highly hierarchical architecture, which can be done with a variety of polymers (Scotti and Dunand, 2018). Since this is a rather well-known and simple approach, it has been widely applied to PNR. Singh et al. (2019) developed an antioxidant polyurethane NGC filled with aligned chitosan-gelatin cryogel filler. Only tested *in vitro*, neonatal DRGs and Schwann cells were seeded on the aligned scaffolds, which resulted in earlier migration and alignment to form “Bands of Bungner”-like structures. Following a stage-wise strategy, Huang L. et al. (2018) produced a directionally freezing oriented collagen-chitosan filler in a porous electrospun PCL conduit. The NGC was optimized to have blend of collagen/chitosan (1:1) as filler and a wall thickness of 400 μm. Such features allowed the NGC to shield growing axons from compression forces while, at the same time adding enough space for regenerating nerves. Furthermore, the anisotropic inner structure allowed Schwann cells and axons from DRGs to extend and migrate parallelly, in a significantly higher rate as compared to a isotropic substrate. Manoukian et al. (2019) took it a step further, and included interconnected longitudinally-aligned pores in a biodegradable chitosan NGC reinforced with drug-loaded halloysite nanotubes. The chitosan conduit allowed the sustained delivery of 4-Aminopyridine, a potassium-channel blocker, that has the capacity to modulate prolonged nerve action potentials and strongly promoted neurotransmitters release. The aligned and interconnected pores allowed for migration of Schwann cells, both longitudinally and horizontally. Furthermore, 4-Aminopyridine delivery resulted in substantial, dose-dependent upregulation of pivotal growth factors, namely nerve growth factor (NGF), myelin protein zero, and brain derived neurotrophic factor (BDNF). Also, it promoted nerve impulse conduction, being an attractive strategy for nerve repair and regeneration.

#### Multichanneled Nerve Guidance Conduits

In order to promote guided nerve tissue regeneration, multichannel NGCs have been used as permissive pathways for axon growth. Microchannels provide both topographical and physical signals within the microchannels of an endoneurium tube, as they imitate and attempt to replace the basal lamina microchannels present in autografts. PLLA nerve conduits, in which each channel diameter ranges 200 μm, showed positive physicochemical characteristics as well as promising neuron oriented differentiation (Liu et al., 2018).

In another study, Hu et al. (2009) describe and tested an original NGC constructed with collagen and chitosan as luminal fillers, in which their inner dimensions approached the ones of the basal lamina microchannels of native nerves. When implanted *in vivo*, the device attained nerve regeneration and functional recovery equal to the positive control, the autograft. PLLA electrospun nerve guides with microchannels were produced by Frost et al. (2018). The conduits walls were covered with fibers for topical guidance and were further evaluated in the rat model of 10 mm sciatic nerve defect. Furthermore, in selected groups, cell transplant derived from autologous stromal vascular fraction was added. The results indicate that electrospun NGCs sustain axonal regeneration *in vivo*. Furthermore, it proves the nanofibers can be used as carriers for transplanted cells.

Using SF biomaterial, Dinis et al. (2015) produced a system containing compact and longitudinally aligned microchannels, representing the fascicles of nerve. Constructed by electrospun SF and capable of incorporating and delivering GFs, the biomimetic multi-channeled functionalized NGCs demonstrated mechanical properties similar to that of rat sciatic nerve. This study suggests that the nerve's native epineurium and perineurium are responsible for the nerve tensile strength.

#### Unidirectionally Aligned Micro- or Nano-Filaments

Unidirectionally aligned micro- or nano-filaments inside NGCs support axonal growth cones in identifying and physiologically answering to the surrounding environment and stimulating them to follow the path to the distal stump. Furthermore, such structures can allow increasing the available surface area to volume ratio, thus potentially enhancing cellular adhesion and proliferation. In one of the first studies of this kind, Matsumoto et al. (2000) added laminin coated fibers as NGC luminal filler, to bridge a 80 mm gap in a canine peroneal nerve model, however with poor outcomes. Quigley et al. (2013) developed an advanced and multi-modal conduit where aligned PLGA fibers are present in the lumen of a knitted PLA sheath coated with electrospun PLA nanofibers. To provide further support, the PLGA fibers are standing on an alginate hydrogel impregnated with several NTFs. The aligned PLGA fibers were remarkably helpful in guiding the growing axons. The fibers formulations was precisely chosen to encourage either axonal outgrowth or Schwann cell growth (75:25 for axons; 85:15 for Schwann cells). Furthermore, axonal outgrowths were found inside and around the NGC and most of the regenerated fibers were positively myelinated. Such type of strategy has been explored in numerous studies, with variable outcomes (Yoshii and Oka, 2001; Yoshii et al., 2003; Kim et al., 2008). What they have in common is that the addition of such filaments clearly extends the regeneration limits when compared to hollow NGCs.

Later, studies were focused on the more suitable diameter of fibers as well as the density of fibers inside the NGC, in order to achieve the best possible outcomes (Jiang et al., 2014; Xie et al., 2014; Jia et al., 2019). Regarding the first parameter, the use of fibers in the “nanometer” scale proved to provide the best outcomes, elevating electrospinning as the technique of choice (Jiang et al., 2014; Jia et al., 2019). Regarding the density of the fibers, lower densities increase the ability to bridge a critical nerve gap (Ngo et al., 2003). Similar to what was found in the case of hydrogels, higher densities and occlusion of the lumen tend to lead to inhibition of regenerating nerves.

#### Magnetically Aligned Cells and Fibers

Magnetically aligned cells and fibers also have valuable effects in PNR. In one of the first studies of its kind, Ceballos et al. (1999) was able to magnetically align type I collagen gel, in which it was visible the nerve fascicle formation. In a similar approach, a study conducted by Rose et al. (2017) where minor quantities of superparamagnetic iron oxide nanoparticles (SPIONs) were combined with the microgels, revealed that the SPIONS could be unidirectionally aligned as long as there was an external magnetic field. In fact, nerve cells could align when seeded in this hydrogel, which is a relevant achievement. Not only magnetically aligned matrices can be valuable, but also aligned cellular components have been developed (Phillips et al., 2005).

Another important technology within the magnetic applications to enhance peripheral nerve regeneration and repair is the use of magnetic nanoparticle to induce cell guidance throughout an organism. For that, magnetic nanoparticles called SPIONs are usually used. For that, Marcus et al. (Marcus et al., 2018) were able to show that neurites would preferentially align along the magnetic field gradient, which was achieved with the help of maghemite SPIONs.

In fact, scaffolds do not need to be magnetically aligned to be deliver positive effects. Liu et al. (2017) produced a scaffold comprising magnetic nanoparticles and a biodegradable chitosan-glycerophosphate scaffolds, which, in combination with a simple magnetic field, was capable of increasing the viability of Schwann cells after transplantation to an animal model *in vivo*. Allied to that, synergistic effects were obtained by combining the magnetic scaffold with a magnetic field, which resulted in enhanced functional recovery after repair of the injury.

#### Micro- or Nanopatterning

In terms of micro- or nano-patterned surfaces for PNR, there are two main approaches that can be used: (i) micro- or nano-patterned two dimensional surfaces that can be rolled up to form a conduit, and (ii) patterned micro or nanomaterials to be used as a filler material in NGCs. A vast selection of studies has used microgrooves to explore the most suitable sizes to encourage axon elongation, having in consideration the cell diameter (Huang C. et al., 2015; Davis et al., 2018; Li G. et al., 2018). Investigational data advocates that thin microgrooves (5–10 μm) confines the growth of cells and axon elongation in comparison to broader grooves (20–60 μm). On the other hand, the narrower microgrooves were found to improve axonal alignment, diminish the number of axon branches per cell, and decrease incorrect distal re-connection. Inclusively, longitudinal nanogrooves (200 nm) sustained functional recovery of sciatic nerves in rats (Huang C. et al., 2015) and enhanced the growth cone attachment and proliferation (Park et al., 2013). Mobasseri et al. (2015) modified the surface topography of PCL/PLA blending films to improve cellular guiding. The results obtained with the sloped walls grooved conduit with 70 μm wall thickness was similar autologous nerve graft, in a gap model of 10 mm in the rat sciatic nerve.

**Figure 6:**
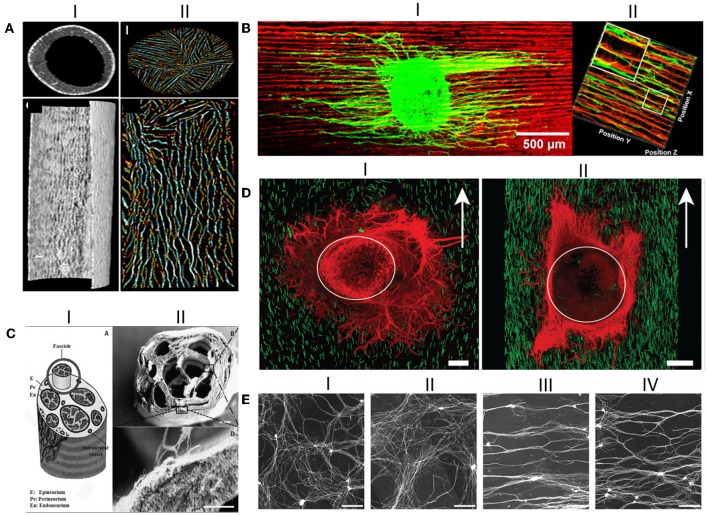
Anisotropic guiding cues have been successfully produced as NGCs luminal fillers. **(AI)** Transverse and longitudinal Micro-CT sections of the hollow conduit; **(AII)** Transverse and longitudinal Micro-CT sections of the oriented chitosan-gelatin cryogel as luminal filler; **(BI)** DRG explants seeded on the longitudinal sections of the directionally orientated collagen-chitosan filler, where neurites align in the matrix; **(BII)** 3D reconstruction of axonal regeneration and Schwann cell migration on the orientated collagen-chitosan filler; **(CI)** Schematic drawing of the peripheral nerve structure; **(CII)** SEM micrograph of the produced silk fibroin NGC fabricated incorporating microchannels, which looks like the depicted schematic; Scale bar 200 μm; **(DI)** DRG explants seeded in 0.25% volume of the anisogel, presenting isotropic structure, in which neurites do not orient; **(DII)** DRGs explants seeded in 1% anisogel in which neurites decide to orient; **(E)** Representative images of DRG explants neurites cultured on random patterns achieved with nanoimprinting lithography with metallic stampers made of three different spacings: **(EI)** on a flat surface; **(EII)** On a Blu-Ray disc spacing; **(EIII)** On a digital video disc spacing; and **(EIV)** On a compact disc spacing. Scale bar: 200 μm. Figures have been reprinted and adapted from: **(A)** (Singh et al., 2019), **(B)** (Huang L. et al., 2018), **(C)** (Dinis et al., 2015), **(D)** (Rose et al., 2017), and **(E)** (Huang L. et al., 2018).

Huang Y.-A. et al. (2018) reported that they were able to exactly place the neuronal cell body and control the direction of axonal growth by merging surface topography and a cell placement device technologies, creating the prospect of engineering complex tissues. Furthermore, they were able to create an on-site axotomy, studying how the topography can influence the initial regeneration of injured axons. Promising results obtained using anisotropic topographical cues as luminal fillers can be seen in Figure 6. However, the incorporation of luminal fillers, regardless of their kind, is not always successful, as a recent study by Saltzman et al. demonstrated (Saltzman et al., 2018). This study aimed to compare the performance of a PGA conduit containing collagen fibers within the conduit, a hollow collagen conduit, and a nerve autograft. The results demonstrated that the nerve repair using the autologous nerve graft resulted in greater motor nerve salvage, followed by the hollow collagen conduit and only then the conduit containing luminal filler. Therefore, a wise selection of the luminal filler must be made since each strategy has its pros and cons.

“The authors of this review, have, however, a criticism on how micro- and nano- scaled features are presented in the literature. Although measurements are identified in micrometers (μm), they are referred to as nanopatterning or nanogrooves. There is very little literature where the nanometer-sized features are explicit in nanometers (nm). Therefore, future works should consider to be more precise in terms of scaling nomenclature.”

#### Allografts

Historically, autologous nerve grafting has been considered the “gold standard” and the most reliable option for nerve repair (Moore, 2014). However, new options have surged with the development of research and with the knowledge that maintaining a 3D structure as luminal filler would result in better functional outcomes after injury reconstruction (Ryan et al., 2017). A strategy that makes use of 3-dimensional cues has been increasingly gaining popularity, being inclusively FDA-cleared (AxoGen®), are the acellular or re-cellularized allografts. Simple allografts from immunologically incompatible donors are not regularly used due to the expense and health risks associated to a life-long of systemic immunosuppression. The solution for such a problematic consists in the extraction of the cellular components, which are and immune-competent.

The definition of processed nerve allografts consists of decellularized human nerves, which keep the internal microstructure and extracellular matrix of native nerve tissue. In fact, processed nerve allografts have been frequently observed to be comparable to the nerve autograft but superior, for instance, to collagen conduits (Moore et al., 2011; Yan et al., 2016; Boriani et al., 2019). The host immune response and functional recovery after nerve injury have also been studied and compared when using autografts or allografts. After studying the immune response 3, 7, 14, 28, and 98 days after grafting autologous or allogeneic nerves without any immunosuppressive treatment, Roballo et al. (Roballo and Bushman, 2019) concluded that while the immune response to autografts is very quick, the response to allografts is slower. It means that the autografts induce a more robust early response, when compared to the slower and gradually adaptative immunological response.

In an allograft, the native ECM is conserved along with the basal lamina, the guiding mechanical and physical cues for axonal regeneration. Several methods can be used to decellularize nerves, among them, physical methods such as lyophilization (Gulati, 1988), direct pressure, and agitation (Freytes et al., 2004). Chemical methods have also been attempted and include digestion with alkaline or acidic solutions (De Filippo et al., 2002), detergents (Woods and Gratzer, 2005), together with the action of enzymes such as trypsin and endonucleases (Gamba et al., 2002). Various studies support the hypothesis that decellularized grafts are among the best options for nerve repair, since they can bridge more than 10–20 mm long gaps in rats (Kim et al., 2004; Lin et al., 2018). However, It has been found, after a systematic review by Boriani et al. (2019) that different processes for cellular components removal deeply affect the capacity to enhance nerve regeneration and leads to differential results in terms of recovery *in vivo*.

## Growth Factors (GFs) as Molecular Therapies

**Figure 7:**
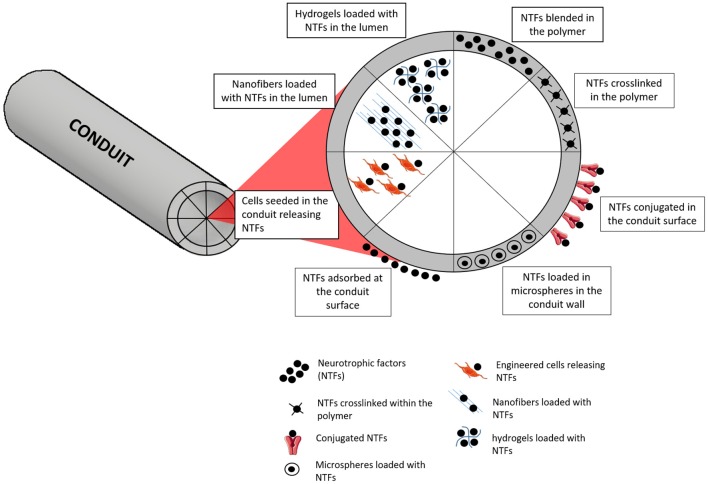
Different strategies for incorporation and delivery of GFs from NGCs. One of the simplest approaches is based on simply blending the NTFs on the polymer, with or without further crosslinking of the polymer. Microspheres containing NTFs can also be blended in the polymer. At the surface, NTFs can be found just after an adsorption process or conjugated with other molecules for stronger entrapment or covalent links. When considering the delivery of NTFs from the lumen, several approaches can be followed, such as using engineered cells, nanofibers or hydrogels capable of loading and releasing the NTFs.

Apart from the use of suitable NGCs and fillers, the conception of a more biologically appealing milieu is of high importance in PNR. Nerve GFs are molecules that are naturally released in the processes of injury and result in enhanced nerve regeneration. Therefore, it is important to mimic their release, which is vital for nerve growth, differentiation and expansion (Tajdaran et al., 2019). However, the artificial administration of GFs as a therapy is problematic duty to accomplish because of their high biological activity, which obliges to administer extremely small doses. Pleiotropic effects and short biological half-life are also other common constraints (Pfister et al., 2007). The fiasco of GF delivery may be credited to unsuitable release kinetics, as several delivery systems unveil an elevated initial burst release (Madduri et al., 2010b). In an attempt to progress in terms of releasing profile, delivery systems that allow adjusting the release kinetics are being investigated (Pfister et al., 2007). The use of biodegradable biomaterials is advantageous in this specific case, since they can act as vehicles for GF delivery, allowing to manipulate specific biomaterials parameters to attain the desired rate of sustained release. Physical crosslinking (Madduri et al., 2010a), chemical immobilization (Aebischer et al., 1989), polymer coating (Madduri et al., 2010a), and nanoparticles (Giannaccini et al., 2017) are some of the strategies that are being used (Figure 7).

The various GFs have diverse actions in the nerve, as they can enhance functional regeneration, support axonal elongation, and Schwann cell migration, by means of acting as neuroprotective components through receptor-mediated activation of specific pathways. GFs regularly used to aid in PNR can be seen in Table 1. NTFs normally used to improve nerve regeneration primarily belong to three separate groups (Deister and Schmidt, 2006): the neurotrophins, the glial-cell-line-derived neurotrophic factor family ligands, and the neuropoietic cytokines. The first group, neurotrophins, include NGF, BDNF, neurotrophin-3 (NT3) and neurotrophin-4 (NT4) (Peleshok and Saragovi, 2006). The second group includes the very glial cell derived neurotrophic factor (GDNF) and the ciliary neurotrophic factor (CNTF) (Rickert et al., 2014). Although they belong to distinct families, all above-mentioned GFs have affinity to different Receptor Tyrosine Kinase (RTKs) on cells, which are trans-membrane proteins that will activate a cascade of events after substrate binding, activating determined cellular responses (Boyd and Gordon, 2003). Furthermore, RTKs are known to be retrograde transported from the extremities to cell body, where they will once again activate transcription and translation of proteins, initiating signaling pathways to boost neuronal outgrowth (Hausott and Klimaschewski, 2016). The GFs of the neurotrophin family are structurally and functionally related peptides with active functions in both CNS and PNS, in particular, they mediate the effective survival and differentiation of several neuronal related cell populations.

**Table 1:** The use of GFs and their effect on CNS and PNS (Terenghi, 1999).

**Neurotrophic factor**	**Neural response**	**Receptor and action**
NGF	Sensory neuron survival Sensory neuron outgrowth Spinal cord regeneration	TrkA/p75, receptors expressed in sympathetic/peripheral sensory neurons (Schwann cells upregulate NGF and p75 in response to PNS injury); Involved in survival signaling and neurite outgrowth;
GDNF	Motor neuron survival Motor neuron outgrowth Sensory neuron survival	GFRa/Ret, receptors expressed in sensory/motor neurons, GDNF primarily produced by Schwann cells in development and plays an important role in sensory regeneration;
BDNF	Motor neuron survival Motor neuron outgrowth Sensory neuron outgrowth	TrkB, BDNF mRNA upregulated in distal nerve stump after sciatic nerve transection; positive modulation of peripheral nerve myelination;
CNTF	Motor neuron survival Motor neuron outgrowth Spinal cord regeneration	CNTFR, present in peripheral nerves and myelinating Schwann cells; promotes survival of motor neurons;
NT-3	Motor neuron survival Motor neuron outgrowth Sensory neuron outgrowth Spinal cord regeneration	TrkC, NT-3 mRNA downregulated in distal nerve stump after sciatic nerve transection; negative modulation of peripheral nerve myelination;
NT4/5	Motor neuron survival Motor neuron outgrowth Sensory neuron survival	TrkB, plays a role in survival of adult sensory neurons;
FGF-2	Motor neuron survival Motor neuron outgrowth Sensory neuron survival Spinal cord regeneration Peripheral nerve regeneration	FGFR1-3 plays a role in regeneration of motor and sensory neurons, as well as in myelination.

### Nerve Growth Factor (NGF)

NGF, the classical member of this family and therefore abundantly characterized, is largely used both *in vivo* and *in vitro*. Its action is limited to a few neuronal cell populations, namely promoting the outgrowth of peripheral sympathetic and neural crest-derived sensory neurons (Shakhbazau et al., 2012a). Recently, Xia and Lv (2018) developed a nanofibrous scaffold loaded with vascular endothelial growth factor (VEGF) and NGF. Although VEGF was only released within the first days, NGF could be continuously released for up to 1 month.

The scaffold capable of dual delivery improved the *in vitro* neural differentiation of induced pluripotent stem cells-derived neural crest stem cells (iPSCs-NCSCs). Additionally, the nanofibrous scaffold was implanted in a serious lengthy defect in rat with positive effects in the both regeneration and vascularization. In addition, recent investigation has revealed that NGF has various effects on inflammatory conditions, beyond the described effects on neuronal cell function (Minnone et al., 2017). NGF effects can be either pro-inflammatory of anti-inflammatory (Mamet et al., 2003). Such is elucidated by the fact that NGF is part of an endogenous mechanism that may have both effects: as it activates immune responses, it also triggers paths essential to inhibit the inflammatory response, reducing tissue injury. However, and since NGF also seems to activate sodium channels and those correlate to the maintenance of inflammatory pain states, the presence of up-regulated NGF is associated to inflamed tissues (Gould et al., 2000).

After injury, it was found that NGF is up-regulated after nerve injury for long periods of time. Curiously, it was also found that peripheral nerve injury triggers a raise in the NGF in the uninjured nerve, on the contralateral side (Shakhbazau et al., 2012b).

### Brain Derived Neurotrophic Factor (BDNF)

As part of the neurotrophin family, BDNF is implicated in learning and memory processes, hippocampal neurogenesis, very important phenomena of synaptic plasticity and is also implicated in nerve regeneration after injury (Lopes et al., 2017).

In peripheral nerves, BDNF is synthesized by Schwann cells, motor neurons, and a specific sub-group of DRG neurons. In fact, after nerve crush or complete transection, BDNF mRNA peaks in all three cell types previously mentioned, including in trkB- and trkC-expressing DRG neurons (McGregor and English, 2019). The up to now known BDNF effects are restricted to certain sub-populations of neurons, including sensory dorsal root ganglion neurons and induction of neurite outgrowth of neurons (Verderio et al., 2006). It has been recently found that BDNF also exerts its effect through stimulation of Schwann cells to produce pro-regenerative cytokines (Lin et al., 2016). In this work, it was demonstrated for the first time that the regenerative stimulation happens through the activation of Janus kinase (JAK)/(signal transducer and activator of transcription) STAT (JAK/STAT) pathway, in Schwann cells, and not on neurons, which was previously considered. Another study (Vögelin et al., 2006) suggested that BDNF may not only stimulate a faster PNR but also significantly can reduce the neuropathic pain, in the rat model.

Regarding BDNF expression and mRNA levels after injury, it was found that there is an up-regulation of BDNF in denervated muscles. Therefore, it implies BDNF is highly produced after an injury to assist in target muscle re-innervation. Furthermore, The levels of such growth factor return to normal after functional recover (Omura et al., 2005).

### Neurotrophin-3 (NT-3)

NT-3 has overlapping neurotrophic activity with NGF, however studies suggest it has an broader specificity as compared to NGF and BDNF (Maisonpierre et al., 1990). Studies show the reinnervation of motor target tissues is also related to the presence of NT-3 and its physiological effects (Sterne et al., 1997). Few reports have been published regarding this growth factor. One of the most recent focuses on the use of NT-3 to overcome Charcot–Marie–Tooth neuropathies, which are a heterogeneous group of peripheral nerve disorders (Sahenk et al., 2014). In these disorders, Schwann cells are affected. However, continued treatment with NT-3 is not feasible due to its short half-life and nonexistence in the market. In a way to overcome this problem, Sahenk et al. (2014) hypothesized that the delivery of NT-3 via gene therapy with an adeno-associated virus. With such strategy and therapy, quantifiable NT-3 amounts were found in blood, in quantities enough to significantly promote nerve regeneration, which was verified by histopathology, and electrophysiology (Sahenk et al., 2014).

Related to the levels of NT-3 after injury, it has been reported in literature that it's levels are keept unchanged after nerve transection (Omura et al., 2005). Interestingly, it has been reported that neurotrophin-4 (NT-4), a prominent indicator for neuron survival, was first found to be up-regulated and then down-regulated in the injured sciatic nerve stumps. Therefore, up-regulation of NT-4 after nerve crush injury might contribute to nerve regeneration (Zhang et al., 2019).

### Glial Derived Neurotrophic Factor (GDNF)

GDNF has an imperative part in the case of degenerative diseases, such as Huntington's and Parkinson's, as it has been found that GDNF encourages survival of damaged midbrain dopaminergic neurons (Cheng et al., 2018). GDNF has also been used to target sensory neurons in order to alleviate pain in cases of chronic denervation (Höke, 2014; Ding et al., 2017). Due to the difficulty to attain the right dosage of released GDNF, most of the recent strategies using GDNF applied to PNR are focused on cellular or gene based-therapies (Shakhbazau et al., 2013; Hsu et al., 2017). In this scope, Shakhbazau et al. (2013) described a proof-of-concept report where Schwann cells were previously engineered with dendrimers or lentiviral transduced with the vector providing doxycycline-regulated GDNF expression. When these GDNF-modified cells were injected into transected peripheral nerves, followed by time-restricted administration of doxycycline, it could be proved that GDNF expression in Schwann cells can be closely controlled and monitored. Using a similar strategy, Hsu et al. created a Cre/loxP-based hybrid baculovirus vector which allowed intracellular formation of episomal DNA minicircle for actual transduction of rat adipose-derived stem cells (ADSCs) and lengthy expression of biologically active GDNF. The implantation of such system into sciatic nerve injury site in rats expressively upgraded nerve repair, which was verified by several parameters, such as enhanced functional recovery, nerve reinnervation, electrophysiological functionality, axon regeneration, myelination, and increased angiogenesis.

Injury-induced upregulation of GDNF expression has been suggested as a tool for nerve repair. Both myelinating and non-myelinating Schwann cells are responsible for the dramatic increase in GDNF expression after injury. GDNF expression is up-regulated after several types of peripheral nerve injury including sciatic nerve crush, axotomy, and compression. It has been reported that GDNF has potent effects on neuronal survival and repair of injured nerves, therefore being a useful therapeutic tool (Xu et al., 2013).

### Gradients of Growth Factors (GFs)

An advanced and upgraded strategy can be considered using NGCs and GFs, which is the use of a GF gradient along with a NGC device. GFs gradient at the surface of the conduit aims at promoting contact guidance along the structure, maintaining the controlled release properties (Tang et al., 2013). This technique is based on the fact that the growth cone at the tip of the axon has path-finding ability, aiming at crossing the gap in a precise direction, correctly reaching the distal target. At the same time which is increasing the speed of such phenomena, what is called growth cone chemotaxis (Mai et al., 2009). Axons extend in search of their appropriate targets, often over long distances with the assistance of growth cones detecting and following molecular gradients (Mortimer et al., 2008).

Many studies have been focusing on this strategy of immobilized concentration gradients, either on films or NGCs, with different approaches (Moore et al., 2006; Yu et al., 2010; Lin et al., 2011; Tang et al., 2013). More recently, Uz et al. (2017) developed a PLLA porous film, that besides having longitudinal surface micropatterns, also presents a gradient of NGF. As a result, the existent surface gradient of NGF contributed to an early fast release from the surface film and enabled oriented neurite outgrowth of PC12 along with the longitudinal micropatterns. With this double strategy, the authors could control the concurrent precise release of neurotrophic factors as well as the directional neurite outgrowth in PC12 cells. In another study (Sun et al., 2019), Sun et al. introduced exogenous cells secreting GFs capable of spatial distribution along the conduit. Instead of using a normal cellular density and distribution along the conduit, the authors used encapsulated bone marrow MSCs (BMSCs) capable of producing high levels of BDNF. In fact, comparing with standard cell lumen injection, the conduits encapsulated with stem cells presented dissimilar cell attachment and distribution after 6 weeks, *in vivo*. Such a construct indorsed Schwann cell relocation from the center to the distal end.

**Figure 8:**
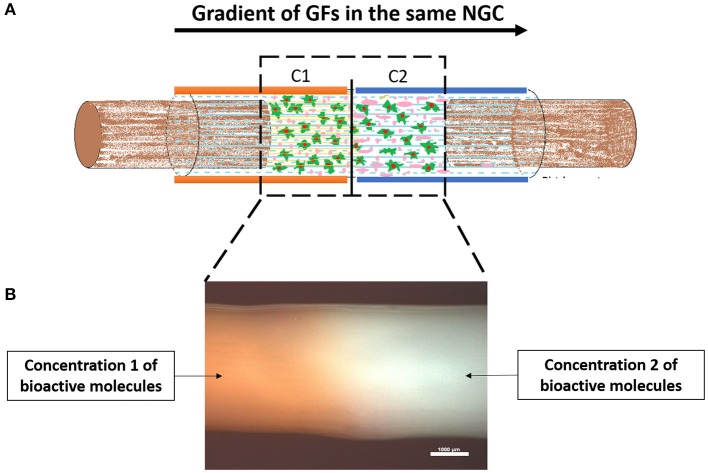
Proof of concept regarding the fabrication of a silk fibroin NGCs incorporating a gradient of GFs. **(A)** Schematic of a NGC incorporating two different concentrations of GFs in the wall of the conduit. In principle, the gradient of GFs increases from proximal to the distal, therefore attracting the growing axons to reach their distal target. **(B)** Stereomicrograph of a silk fibroin NGC presenting a gradient along the walls. The orange color represents the chosen Concentration 1, followed by the white color, representing Concentration 2. As it can be assessed, there is no separation in the conduit between the different concentrations, as the conduit is totally uniform. Scale bar: 1,000 μm.

In another interesting and combinatorial approach, Chang et al. (2017) developed a natural biodegradable multi-channeled scaffold composed by oriented electrospun nanofibers containing a neurotrophic gradient. For the gradient, the authors followed two strategies: the NGF was simply blended with gelatin and BDNF was encapsulated in nanoparticles further embedded in gelatin hydrogel. The gelatin scaffold was divided into five regions from A1–A5 (low concentration to high concentration, from proximal to distal site). Their strategy promoted intense nerve regeneration in critical sciatic nerve defect in a rabbit model. Our group has also been developing a GF gradient strategy, by fabricating silk fibroin NGCs containing GF gradients in its walls, as can be seen in Figure 8. However, unlike Tang et al. (2013), who simply used soaking and coating techniques in the silk fibroin conduits, our group's strategy is based on gradually entrapping the GFs in the conduit's wall. This is achieved by enzymatically crosslinking the polymer, mediated by a horseradish peroxidase/hydrogen peroxide reaction.

## Other Biological Cues

### VEGF

Not only the three major groups of neurotrophins exerts positive effects on neuronal regeneration. One such growth factor is VEGF. As aforementioned, peripheral nerves comprise numerous packs of axon fibers and blood vessels, which are further enclosed by connective tissue. Furthermore, it has been noticed by scientists that the vascular and the nervous system show similar anatomical structures, revealing a strong interconnection between both systems (Andreone et al., 2015). Besides that, ischemia and oxygen deficiency occur in the injured nerve, as well as the destruction of the blood-nerve barrier. Despite the top most position of VEGF as a pro-angiogenetic factor, a collection of reports focuses the attention on VEGF activity on its neurotrophic and neuroprotective effect, both *in vitro* (Sondell et al., 2000) and *in vivo* (Hobson et al., 2000). The administration of VEGF can result in earlier functional improvement of the sciatic nerve when compared to the controls, since higher nerve neo-vascularization leads to improved nerve morphology (Mohammadi et al., 2013a). More recently, it also was discovered through *in vitro* assays that the addition of VEGF to primary Schwann cells promotes their migration, a major process in the promotion of neurite outgrowth (Muratori et al., 2018). Interestingly, VEGF-B has been tested on injured nerves despite its lack of angiogenic activity, but because of its neuroprotective effect, making VEGF-B an appropriate therapeutic agent to administer in the case of nerve injury (Guaiquil et al., 2014; Calvo et al., 2018).

### Hepatocyte Growth Factor (HGF)

In a work developed by Ko et al. (2018), hepatocyte growth factor (HGF) has been recently found to be up-regulated, resulting in a higher expression of the referred HGF after a PNI, both at the injury and distal sites. The authors tested this specific growth factor, with known angiogenic activity and anti-inflammatory activity (Nakamura and Mizuno, 2010) in a model of PNI in mice. After the injury, not only HGF was highly expressed, but its receptor, c-met, was also found to be upregulated only in Schwann cells. In addition, exogenous administration of HGF at the injury site led to an increase of the myelin thickness and axon diameter in injured nerves. To further prove this fact, when mice were treated with a c-met inhibitor, the opposite happened, as myelin thickness and axon regrowth were diminished, indicating that the positive effect of HGF was hindered. In line with this findings, Boldyreva et al. (2018) also studied the effect of HGF in PNR. Gene therapy with HGF-bearing plasmid (pC4W-hHGF) led to the repair of nerve morphometry and functional recovery comparable to the autograft (positive control). Moreover, in HGF-treated mice, histological evaluation showed a three-fold intensification in axon quantification in the distal nerve end, when compared to control, indicating great potential in keeping a healthy distal target. Besides confirming the potential of the application of HGF in cases of PNI, gene-therapy itself proved to be effective and advantageous. In addition to confirmed beneficial effects in PNR, literature has revealed that this growth factor applies beneficial effects in motor, sensory, and parasympathetic neurons. Furthermore, the beneficial effects of this growth factor can be considered mitogenic, morphogenic, angiogenic, antiapoptotic, antifibrotic, and anti-inflammatory, which can act upon numerous tissues (Imamura and Matsumoto, 2017).

### MicroRNA

MicroRNAs (miRNA) are minor endogenous non-coding RNA molecules capable of regulating of gene expression after transcription. There are ~23 of these molecules, that control the expression of several genes (Bartel, 2009). The role of miRNAs may go overlooked. However, at a post-transcriptional phase, it is projected that miRNAs control around 60 % of the total human genes, therefore playing critical roles in cell differentiation, proliferation, migration, apoptosis, and morphogenesis (Rana, 2007). Related to nerve injury and regeneration, studies have been shown that miRNA play an important role in neuronal disease, as it has been reported that a global deregulation of miRNAs occurs in cut sciatic nerve axons (Li et al., 2013). More specifically, it was found through *in vivo* and *in vitro* studies that the removal of Dicer (a crucial molecule in biogenesis of miRNA) disturbs the creation of Dicer-dependent miRNAs, consequently impeding PNR. Such findings confirmed the importance of Dicer-dependent miRNA pathway for effective repair of nerve injuries (Eacker et al., 2009). Bremer et al. (Bremer et al., 2010) reported that numerous miRNAs, including miRNA-34a, miRNA-146, miRNA-30a, miRNA-195, miRNA-140, miRNA-27b, and miRNA-204, were upregulated in the phenomena of myelination in Dicer mutant mice. On the other hand, Gokey et al. (2012) revealed that 225 miRNAs were present during myelination. By its turn, miRNA-106a, miRNA-20b, miRNA-338, miRNA-92b, miRNA-19b, miRNA-363, miRNA-350, miRNA-17, and miRNA-340 are capable of controlling Sox10, adjusting myelin genes, and having a direct impact on myelination.

**Figure 9:**
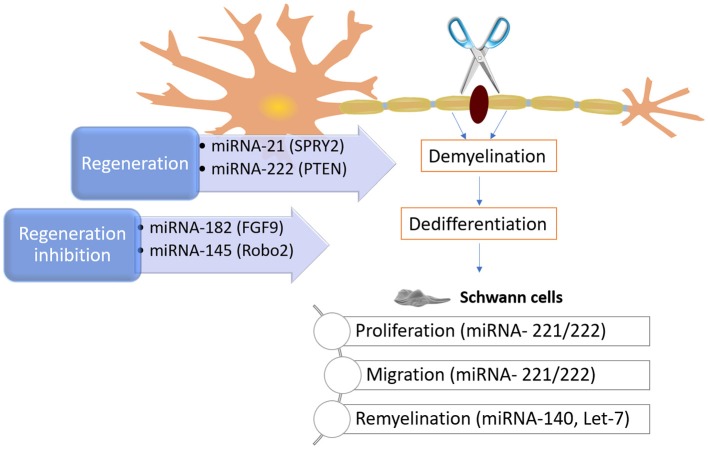
Schematic on some known mechanisms of how miRNAs can intrinsically control and impact peripheral nerve injury and regeneration. After an injury, the myelin and axons degrade, and Schwann cells dedifferentiate. As these phenomena happen, the molecular regulators (e.g., miRNA-221, miRNA 222, and Let-7) can influence neurite outgrowth and modulate phenotypic changes in Schwann cells, as well as their myelinating capacity, among others.

The importance of miRNA is also supported by the fact that Schwann cells proliferation and migration are controlled by let-7, by controlling NGF expression. The decrease of let-7d encouraged Schwann cells to intensify NGF expression, leading to axon regrowth (Li et al., 2015). As mentioned, let-7 miRNAs are extremely plentiful during the myelination process. However, as antagonists, their levels are contrariwise associated to the expression of lin28. Small amounts of let-7 miRNAs are the consequence of continuous expression of Lin28B, which lead to poor Schwann cell myelination (Gökbuget et al., 2015). The influence of miRNAs was also verified on neurite outgrowth from DRGs after nerve injury. In one hand, miRNA-21 encouraged neurite outgrowth by downregulating Sprouty2 expression (Strickland et al., 2011). On the other hand, miRNA-222 targeting PTEN promoted neurite outgrowth. In contrast, the Robo2 expression can be inhibited by blocking the miRNA-145, subsequently reducing neurite outgrowth (Zhang et al., 2011). The knowledge about the mechanisms involved and controlled by miRNAs offers the opportunity to explore potential new therapies, at a molecular level. Some of the mechanisms and miRNAs involved in PNR can be seen in Figure 9.

## Cell-Based Therapies

The importance of cells as crucial actors in the nerve regenerative process is evident. Consequently, cell-based therapy became a significant and evidenced intervention which improves the functional clinical outcome after nerve injury (Yousefi et al., 2019). Many approaches can be followed, from primary Schwann cells, neural stem cells, embryonic stem cells, bone marrow stromal cells (BMSCs) and TE cellular components. Some of the most recent and relevant published reports, where cell-therapies are an important tool for PNR, regarding the use of several cell types can be seen in Table 2.

**Table 2:** Cell-based therapies for PNR.

**Cell type**	**Defect size, location, animal model**	**Outcomes**	**References**
Schwann cells	15 mm, sciatic nerve, rat	Schwann cells overexpressing FGF-2 in a chitosan conduit supported the early regenerative process;	Meyer et al., 2016a
	5 mm, bilateral cavernous nerves, rat	Simple Schwann cells or GDNF-transduced Schwann cells grafts led to 75 % and 94 % success rate, respectively, compared to the 25 % of autografts;	May et al., 2016
	*In vitro*, micro-patterned surface fabricated by laser ablation with NGF	When co-culturing with Schwann cells, NSCs differentiated into neuronal cells with robust expression of βIII tubulin and microtubule-associated protein-2;	Yeh et al., 2017
	5 mm, laryngeal nerve, rat	Laminin-chitosan-PLGA NGC combined with Schwann and NSC promoted significantly higher nerve regeneration when compared to acellular grafts;	Li Y. et al., 2018
	*In vitro* co-culture of Schwann cells and DRGs	Schwann cells in co-culture with DRGs promoted longer neurite extension and formation of myelin around DRG neurites;	Wu et al., 2018
Bone Marrow stem cells (BMSCs)	20 mm autograft, sciatic nerve, rat	BMSCs can differentiate into Schwann cell-like phenotype and myelinate axons, also expressing neuronal markers such as GFAP and S100;	Keilhoff and Fansa, 2011
	10 mm, sciatic nerve, rat	Tropomyosin receptor kinase A overexpression enhanced the efficacy of BMSCs on PNR and improved functional recover;	Zheng et al., 2017
	Contusion injury of the spinal cord, rat	Intravenous delivered BMSCs exosomes tend to migrate into the injury site, where they exert their beneficial effects;	Lankford et al., 2018
Undifferentiated adipose derived stem cells (ADSCs)	10 mm, sciatic nerve, rat	Number and diameter of the myelinated fibers were significantly higher in the case of silicone NGC loaded with ADSCs;	Santiago et al., 2009
	6 mm, sciatic nerve, rat	Decreased muscular atrophy and enhanced PNR when PCL conduits were loaded with ADSCs;	Mohammadi et al., 2013b
	Blunted injury, sciatic nerve, mouse	Transplanted ADSCs did not differentiate into Schwann cells but promoted PNR, since they encouraged axon regeneration, formation of myelin and restoration of denervated muscle atrophy;	Sowa et al., 2016
	15 mm, sciatic nerve, rat	ADSCS injected directly in the muscles connected to the damaged nerve were found to have increased presence of IL−10 and Ki67, which helped in delaying the onset of muscular atrophy;	Schilling et al., 2019
Differentiated adipose derived stem cells (ADSCs)	10 mm, sciatic nerve, rat	Schwann cell-like differentiated ADSCs were found to express neurotrophic factors, namely NGF, BDNF, glial-GDNF, and NT4. The same study also reported an increase of anti-apoptotic m-RNA of Bcl-2 as well as a decrease of pro-apoptotic m-RNA Bax and caspase-3, which lead to a neuroprotective state;	Reid et al., 2011
Human umbilical-cord stem cells (HUCMSCs)	10 mm, sciatic nerve, rat	HUCMSCs increased the expression of neurotrophic and angiogenic factors, which led to a more favorable environment for nerve regeneration;	Shalaby et al., 2017
	10 mm, sciatic nerve, rat	Wharton jelly-derived stem cells, in addition to an injection of dexamethasone resulted in advanced regeneration compared to the autograft;	Moattari et al., 2018
Olfactory ensheathing cells (OECs)	8 mm, sciatic nerve, rat	PLLA NGC seeded with OEC encouraged nerve regeneration similarly to the autograft group;	Kabiri et al., 2015
	*In vitro*, to test how OECs promote neurite outgrowth of cortical neurons in an inhibitory scar-like culture model	It was found that OECs enhanced neurite elongation through direct contact and alignment of neuronal and OEC processes in scar-like cultures;	Khankan et al., 2015
	5 mm, facial nerve, rat	OECs transplanted within the NGC improved regeneration of transected facial nerve, with large numbers of myelinated nerve fibers, crude fibers, larger myelin thickness and volume in the transplanted graft;	Gu et al., 2019
Neural stem cells (NSCs)	Intra-orbital crush, optic nerve, mouse	Intravitreally grafted NSCs differentiated into astrocytes that survived in the host eyes, stably expressed CNTF and significantly attenuated the loss of the axotomized retinal ganglion. The CNTF-secreting NSCs also induced long-distance regrowth of the lesioned retinal ganglion axons;	Flachsbarth et al., 2014
	3 mm, sciatic nerve, mouse	The addition of IL12p80 together with NSCs in NGCs improved motor function recovery, promoted nerve regeneration and increases the diameter of newly regenerated nerve up to 4.5 fold.	Lee et al., 2017
Skin-derived precursors (SKPs)	10 mm, sciatic nerve, miniature pigs	SKPs transplantation showed better *in vivo* nerve regeneration potential than in the non-cell transplantation control group, with increasing expression of S100 and P75NGFR;	Park et al., 2012
	Cutaneous nerve regeneration, 1 × 1.5 cm^2^ circular island of skin, mouse	SKPs were found to be neurotropic toward injured nerves. They had a full capacity to differentiate into Schwann cells and promote axon regeneration. SKPs revealed to be an active participant in cutaneous nerve homeostasis;	Chen et al., 2012
	15 mm, sciatic nerve, rat	The addition of Schwann cell – like SKPs increased sciatic nerve functional index, peak amplitudes, nerve conduction velocities, number of myelinated fibers, and decreased muscle atrophy;	Wang et al., 2016
Genetically modified cells	10 mm, sciatic nerve, rat	The transfected cells secreted GDNF at higher rate which enabled better survival of motor neurons when compared to controls. Furthermore, there was an enhanced expression of GDNF mRNA;	Li et al., 2006
	15 mm, sciatic nerve, rat	FGF-2 overexpressing Schwann cells were seeded in a chitosan film inside a chitosan conduit, which enhanced nerve regeneration;	Meyer et al., 2016b
	End-to-end suture, sciatic nerve, rat	GDNF-expressing ADSCs revealed a robust expression of GDNF throughout time, where regeneration of nerve was significantly improved as evidenced by enhanced functional recovery, nerve reinnervation, Schwann cell migration and proliferation, axon regeneration, myelination, and angiogenesis;	Hsu et al., 2017
	10 mm, sciatic nerve, rat	KLF7-transfected Schwann cells enhanced motor and sensory axonal regeneration. Myelinated fibers were also significantly higher;	Wang Y. et al., 2017

### Schwann Cells

Schwann cells play a critical part of the Wallerian Degeneration. The high number of Schwann cells after an injury, seventeen times more than seen in the uninjured nerve, proves they are activated, and their presence is beneficial in case of injury (Evans et al., 2002). Therefore, many therapies are based in the transplantation of autologous or allogenic Schwann cells. Once expanded and harvested, Schwann cells will help in PNR after introduced in the NGC, which can be attained by a variety of methods: direct injection of a cellular suspension to the lumen (Jesuraj et al., 2011), suspension within an intraluminal hydrogel (Cerqueira et al., 2018), distributed along intraluminal guidance structures (Meyer et al., 2016a) or released from the luminal (Kalbermatten et al., 2008). It has been considered that autograft is the gold standard of PNR and autologous Schwann cells are the gold standard of cellular-based therapies (Pearse et al., 2018). However, the use of Schwann cells comes with a few shortcomings such as the effort of collection, the time-consuming expansion in culture and a inappropriate immunogenicity, thus requiring further immune suppression strategies to be considered. Due to these drawbacks, the attention shifted to the use of undifferentiated stem cells, which can differentiate into several cell types in the presence of specific drugs/growth factors.

Stem cells can be obtained from many sources, but most studies focus on the use of both BMSCs (Oliveira et al., 2013) and ADSCs (Zhang and Rosen, 2018). For TE purposes, they have ideal properties such as differentiating in multiple lineages, being simply extracted, proliferating quickly in culture, having cheap maintenance and above all, do not raise ethical issues (Wang C. et al., 2017).

### Bone Marrow Stromal Cells (BMSCs)

BMSCs are one of the most important types of stem cells. Their primary function is to support hematopoiesis in the stromal compartment of bone marrow, from where they are withdrawn with a simple process of aspiration, after which they are easily expanded (Abdallah and Kassem, 2008). Many studies have focused on learning the mechanisms through which BMSCs are beneficial for PNR (Mimura et al., 2004; Ishikawa et al., 2009; Hou et al., 2018). Xu et al. (2011) developed an *vitro* study in 2011 which demonstrated that a co-culture of BMSCs and DRGs explants stimulated neurite growth and neuronal cell survival through the up-regulation of wide list of secretory proteins, including bFGF, NGF, CNTF, and BDNF. Also, by means of using a co-culture system of DRGs and BMSCs in a trans-well, it was found that the presence of DRGs helped keep the stemness of BMSCs using the AMPK/mTOR signaling (Zhang et al., 2018).

In an *in vivo* model of long-gap transected sciatic nerve in adult rats (Mimura et al., 2004), BMSCs-derived Schwann cells were firstly suspended in Matrigel, followed by injection within the conduit in the injury site. The results demonstrated regenerated axons in the central portion of the graft, which was significantly greater in the transplanted group. The study further demonstrated that BMSCs did not differentiate in any other kind of cells, and tumor formation did not occur. In a more recent study (Zheng et al., 2017), rat BMSCs were genetically modified with recombinant lentiviruses to construct TrkA-overexpressing BMSCs. They were then seeded in acellular nerve allografts to bridge 10-mm rat sciatic nerve defect. Eight weeks after surgery, the analyses demonstrated improved axon growth, as well as significantly higher expression of myelin basic protein and superior results of myelinated fiber density, axon diameter and myelin sheaths thickness, revealing a superior outcome in terms of nerve regeneration.

### Adipose-Derived Stem Cells (ADSCs)

ADSCs isolated from adipose tissue are one of the most used stem cells. Many *in vitro* and *in vivo* studies have confirmed the ability of ADSCs to promote nerve regeneration, in both undifferentiated (Zhang and Rosen, 2018) and differentiated (Ching et al., 2018) conditions. Concerning undifferentiated ADSCs, the main concern is related to the possible differentiation that cells may undergo when implanted, originating non-desirable phenotypes, such as adipocytes. In fact, nerve regeneration may be delayed due to fat obstructing NGCs (Papalia et al., 2013). In respect to Schwann cell-like differentiated ADSCs, they were found to express a variety of intrinsic neurotrophic factors, namely NGF, BDNF, GDNF, and NT4 (Reid et al., 2011).

### Human Umbilical-Cord Stem Cells (HUCMSCs)

Human umbilical-cord mesenchymal stem cells (HUCMSCs) are also becoming very popular in TE and more specifically in PNR purposes. These cells display exceptional characteristics to be use in TE approaches, such as multilineage potential, immunomodulatory aptitude, effortless isolation, and fast proliferation (Ding et al., 2015). Besides, 14 different neurotrophic factors related to enhance PNR are secreted by HUCMSCs (Guo et al., 2015). These factors stimulate neuronal survival, vascularization, upregulation of cell binding integrins, delivery of anti-inflammatory molecules, and increased survival and proliferation of Schwann cells. Thus, HUCMSCs paracrine effects probably lead to the efficacy of those cells in the treatment of nerve injuries (Ma et al., 2019).

### Olfactory Ensheathing Cells (OEC)

Still not widely recognized by the scientific community, Schwann cells and olfactory ensheathing cells (OECs) are two types of glial cells with crucial performance in reestablishing function and sensation after nerve injury within the PNS. OECs have similar capacities to Schwann cells, as they can reduce scar formation and promote regeneration (Yao et al., 2018). As a component of both PNS and CNS, reports on the use of OEC have mostly been focused on the CNS and insufficient assays have addressed their worth for PNI. One of the mechanisms by which OECs assist in PNR, is that they produce neurotrophins, such as NGF, BDNF, NT-3, NT-4/5, BDNF, and GDNF, leading to neuronal outgrowth and axonal regeneration (Woodhall et al., 2001). However, unlike Schwann cells, OECs are not capable of producing cytokines that attract macrophages (Su et al., 2009).

### Neural Stem Cells (NSCs)

NSCs have also been used for PNR. Isolated, among others sources, from the adult striatum, they have the remarkable ability to divide, proliferate, and experience multi-lineage differentiation *in vitro* (Tang et al., 2017). This fact eliminates the pre-conceived theory that neural tissues cannot regenerate (Wang C. et al., 2017). Several studies have proved the capability of NSCs to differentiate into Schwann cell-like phenotypes, being positively marked for anti-S100 and anti-p75 and being capable of producing a myelin sheath (El Seady et al., 2008; Tong et al., 2010).

### Skin-Derived Precursor Cells (SKPCs)

The dermis in the skin comprises neural crest related precursor cells, namely the skin-derived precursor cells (SKPCs). They can be differentiated into different cell types belonging to the neural crest, such as neurons and Schwann cells, to find their application in the PNS (McKenzie et al., 2006). For such, SKPCs are cultured in medium enriched with neural crest cues such as neuregulins, after which SKPCs morphology switches to Schwann cells-like. These cells are capable of inducing myelin proteins transcriptions when in contact with neurites (McKenzie et al., 2006).

### Genetically Modified Cells

Genetically modified cells have been extensively used in the scope of TE strategies, inclusively for PNR. To be applied in the PNS, modifications are carried out in primary Schwann cells. However, the use of genetically engineered MSCs is also common (Yousefi et al., 2019). Genome editing is a remarkable expertise in which therapeutic and interesting genes are inserted in specific cells with the goal of reprograming the cells to express or inhibit certain genes of interest. The main objective of gene alteration in this field is reprograming cells to over-release GFs, migratory and adhesive molecules as well as hindering un-desired or negative effect-related genes (Jandial et al., 2008). In what concerns the molecular biology tools used to genetically modify cells, Herpes and recombinant virus are the typical used vectors in TE, as they are associated to high yields. However, comparing with traditional viruses, the recently discovered AAV-PHP.eB and AAV-PHP.S derived from adeno-associated viruses presented better outcomes, being able to transduce PNS cells (Chan et al., 2017). One basic purpose of genetically modification of Schwann cells is for them to produce green fluorescent protein (EGFP), in order to follow their fate and track their role in nerve repair and myelination. Many studies use this methodology (Schmitte et al., 2010; Deng et al., 2014; Zhang et al., 2017). With the intuit of overexpressing a specific GF, Timmer et al. (2003) implanted a silicon tube filled with Matrigel with enclosed transfected Schwann cells to overexpress fibroblast growth factor (FGF-2). The transfected cell group revealed an intensification in the number of myelinated axons as compared to the control group. To surpass the “candy store effect,” Marquardt et al. (2015), used Schwann cells that were previously transduced with a tetracycline-inducible GDNF expressing lentiviral vector. Consequently, it allowed the expression of GDNF to be temporally controlled by doxycycline administration. Also, within the PNS, the intracochlear injection of GFs using mini-osmotic pump has been shown to avert deafness-induced spiral ganglion neuron degeneration. However, it is associated with infections in the cochlea, making it inappropriate to use this method in clinics. Therefore, Pettingill et al. (2008) used gene-based therapy as an alternative. In this study the authors used Schwann cells modified to express higher levels of BDNF or NT-3. The results suggest that that this therapy was useful in enhancing survival of spiral ganglion neurons.

However, in 2015, Huang L. et al. (2015) made the incredible discovery that by genetically modifying Schwann cells to overexpress c-Jun, a constituent of the AP-1 transcription factor, it would successfully upregulate the production of multiple GFs, including NGF, GDNF, BDNF, artemin, and leukemia inhibitory factor. A single modification capable of altering the whole process of nerve regeneration and myelination is a potential therapy, that has shown outstanding results.

After reviewing in detail the most recent and relevant literature related to the three main pillars of Tissue engineering and Regenerative Medicine, namely 3-dimensional scaffolds as intraluminal guiding cues, several cellular components as well as diverse growth factors, it is the authors opinion that a combinatorial approach should be the most fruitful. In one hand, the scaffolds or polymeric materials mimic ECM, highly important for endogenous cellular attachment and proliferation, leading to a tissue remodeling. For instance, NeuraGen 3D® nerve guide matrix by Integra Lifesciences presents itself as a very promising and clinically available 3-dimensional scaffold (Lee et al., 2012; Mobini et al., 2017). However, growth factors, which can and should be used synchronously with biomaterials, are the chemical components or signaling biomolecules, that direct the fade of the cells by instructing them in phenomena such as adhesion, proliferation, or differentiation (De Witte et al., 2018). According to clinical trials, scaffold-based growth factor delivery systems are the ones with auspicious results (Lee et al., 2011), since it allows a proper spatio-temporal control over the location, amount, and bioactivity of the released factors.

In what regards the addition of cellular components, although the reviewed reports usually suggest its use is beneficial for PNR, the regulation/legislation and possible commercialization of medical devices containing cells is of very difficult approval, due to the lack of reproducibility and high variability. Is the authors opinion that acellular off-the-shelf products are more suitable and easily approved for the process of clinical translation, which is urgent at the moment.

## Neuroplasticity

Neuroplasticity plays a chief role in PNI patients and how their recovery therapy is carried. Briefly, the term neuroplasticity refers to the ability of the neuronal system to change (Navarro et al., 2007). It is now recognized that the brain is not a hard-wired unchanged circuit, but an adaptive system that may experience injury-induced changes. Research in this field has already proved and continues to deepen the available information regarding the interplay between the CNS and PNS throughout an entire lifespan (Taylor et al., 2009). Animal experiments have recognized that the so called neuroplasticity in the cortex begins instantly after peripheral nerve injury (Wall et al., 1986). More recently, resourcing to advanced imaging techniques, many reports established that a PNIs are linked to quantifiable structural changes in the human brains (Seil, 2014; Makin and Bensmaia, 2017; Nordmark et al., 2018). For instance, the persistent and chronic pain often present in PNI patients' leads to physical changes in the brain, such as insula thickness (Goswami et al., 2016). In another example, functional magnetic resonance image (fMRI) has shown that patients who had neuromuscular interventions control the reconstructed muscle with different parts of the brain when compared to the healthy muscle (Chen et al., 2003).

After peripheral nerve transection and surgical repair, it has also been noticed that both gray and white matter suffer from physical and organizational irregularities in numerous cortical areas as well as thinner cortex, revealing the functional plasticity (Taylor et al., 2009). The literature concentrates mainly on measurable parameters and the progress of modern non-invasive neuroimaging techniques to scrutinize brain plasticity in humans. Among the first category, nerve conductions studies, quantitative sensory testing and neuronal responsiveness to stimulus are evaluated (Oberman and Pascual-Leone, 2013). Related to neuroimaging, fMRI is capable of measuring white/gray matter volume, density and integrity, as well as cortical thickness (Zatorre et al., 2012).

Until today, in the clinical setting, the standard diagnosis as well as treatment of PNI patients' relies solely on the fusion of simple clinical examination as well as neuro-electrophysiology measurements (Tagliafico et al., 2010). Consequently, the analysis accuracy is frequently decreased since the two approaches mentioned before do not provide adequate information for the needed surgical repair or treatment. Several imaging techniques exist, such as diffusion tensor imaging (DTI), ultrasound and positron emission tomography (PET) (Rangavajla et al., 2014). However, assessments by fMRI usually achieve the highest resolutions and are capable of delivering detailed information and necessary quantifications (Rangavajla et al., 2014). This technique can, complimentary to the classical evaluation methods that give little information, provide satisfactory spatial and temporal resolution for evaluation of degeneration or regeneration after injury for a correct diagnosis or treatment.

Not only physical changes can be detected, but physiological and emotional traits can also be recognized, such as depression or the phenomena of catastrophizing pain (Fernández-Muñoz et al., 2016). Maladaptive neuroplasticity also occurs very often. Chronic neuropathic pain is the result of pain-related changes as the answer to the injury (Jensen and Finnerup, 2014). Although not fully understood, it is suspected that neuroplasticity arises from deficient and incomplete nerve regeneration, to which the brain must physically adapt. Cortical changes following PNIs include topographic restructuring of the somatosensory and motor cortices, which will be long lasting.

**Figure 10:**
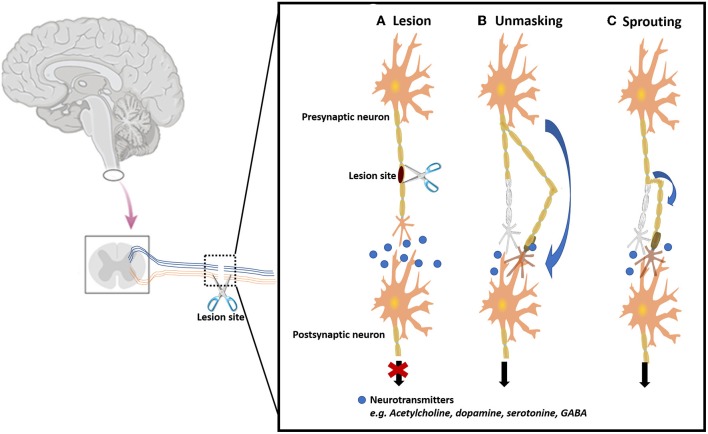
Scheme representing the neuroplasticity that occurs throughout the CNS after PNIs. **(A)** Healthy peripheral nerve being subjected to an injury. Immediately after the trauma, the functionality of the nerve is affected, and the correct neurotransmission is interrupted. Neuroplasticity that occurs in the CNS following PNI is thought to occur through several mechanisms, with two of the most prominent theories being: **(B)** Unmasking of existing synaptic connections. In this process, there is the unmasking of neural paths which were not normally used for a specific purpose, and new neural paths are activated when the normally used system fails; and **(C)** Sprouting of new nerve terminals, where there is collateral sprouting from intact healthy cellular components to a denervated region, in an attempt to reestablish the neuronal connection.

Overall, the importance of this specific field relies on the fact that considering peripheral and central changes that directly affect the patient recovery may lead to the advance of new healing and therapeutic approaches, including tailoring of timing and rehabilitation policies (Oberman and Pascual-Leone, 2013). A scheme representing neuroplasticity can be seen in Figure 10.

## Clinical Trials in the Scope of PNR

Several pre-clinical studies have been carried by the scientific community along the last decades and have proven the value of innovative therapies and discoveries in the case of PNR. The proof are the several hundreds of reports that have been published in the niche of this scientific area. These do not correlate to the very scarce clinical trials associated to PNR, found in the platform ClinicalTrials.gov.

More specifically, a brief research was made in the PubMed platform, in an attempt to find a relative number indicating the pre-clinical trials (academic studies/manuscripts) that have been published related to “peripheral nerve regeneration” in the last decade (2009–2019). 3439 studies were found, revealing the extensive research and funding that has been invested in this neurological field, in the Academia. However, only 356 clinical trials were ever performed, when searching the same words in the platform ClinicalTrials.gov.

On the other hand, even though a few clinical trials have found and reported in this review (Table 3), these clearly outnumber the strategies and policies currently available in the clinics for the patients. Such facts are a worrying reason, since no real effort seems to be being made to take the diverse explored alternatives to the actual patients in need. However, after a brief research in the ClinicalTrials.gov platform, a few categories could easily be found related to strategies that are, or have been, tested in humans in attempts to treat PNIs. Electrical stimulation (ES) is the first category analyzed. It is not uncommon to find studies assessing the benefits of ES, since it has been shown that ES accelerates axonal regeneration in the case of an injury. Undoubtedly, PNS relies on electrical activity to carry its basic functions and pre-clinical studies have unquestionably shown its usefulness (Gordon and English, 2016; Willand et al., 2016; MacEwan et al., 2018). In this scope, different tactics are being tested in humans, *e.g*. ES pre-operatively (NCT03205124), extra-corporal ES after nerve coaptation (NCT03147313) or immediately after surgery NCT02403661.

**Table 3:** Clinical trials in the scope of PNR being carried in different countries and with different strategies.

**Strategy or technology used**	**Name of the clinical trial study**	**ClinicalTrials.gov** **Identifier**	**Sponsors and locations**	**Specifics of the study**
Electrical stimulation	The Effect of Pre-operative Electrical Stimulation on Peripheral Nerve Regeneration	NCT03205124	University of Alberta	Patients randomized to this group will receive 1 h of continuous electrical stimulation 3 days prior to scheduled surgical date;
	Electrical Stimulation to Enhance Peripheral Nerve Regeneration	NCT02403661	University of Alberta	The goal is to test the possible benefit of electrical stimulation of the injured nerve following surgery;
	Extra-corporal Shock Wave Treatment to Improve Nerve Regeneration	NCT03147313	Meidling Trauma Hospital, Lorenz Böhler Trauma Hospital	This study evaluates the impact of extracorporeal shock wave treatment after microsurgical coaptation of finger nerves;
TE NGC or other biomaterial approaches	Mid-term Effect Observation of Biodegradable Conduit Small Gap Tubulization Repairing Peripheral Nerve Injury	NCT03359330	Peking University People's Hospital	The biodegradable conduit small gap tubulization are used to repair the nerve. Their nerve functional recovery conditions are clinically observed according to the standard score methods;
	Prospective Analysis of Effect of Collagen Wrap Conduit on Radial and Ulnar Nerve Function Following Radial/Ulnar Forearm Free Flap Harvest	NCT03875833	The University of Texas Health Science Center	Determination whether collagen nerve conduits placed on exposed radial and ulnar nerves during radial and ulnar forearm free flap harvests will reduce the occurrence and degree of sensory nerve deficit;
	Evaluate the Reconstruction of Digital Nerve Defects in Humans Using an Implanted Silk Nerve Guide	NCT03673449	UniversitätsSpital Zürich,Recruiting, Zürich	Ascertain the feasibility and safety of the procedure using SilkBridge—a biocompatible silk fibroin-based scaffold—for the regeneration of sensory nerve fibers and follow it up together with the reinnervation of the target organs;
	Clinical Study for the Treatment of Peripheral Nerve Defects with Neuromaix (PeRepair)	NCT01884376	RWTH Aachen University	The aim of this study is the development and initial clinical application of the nerve guide Neuromaix in humans to provide evidence for the safety and performance of the device;
	A Phase I Trial of a Novel Synthetic Polymer Nerve Conduit 'Polynerve' in Participants with Sensory Digital Nerve Injury (UMANC)	NCT02970864	University of Manchester	Participants found to have a nerve gap of at least 5 mm and no >20 mm will undergo repair with the Polynerve. Participants will be followed up regularly, observed for device-related complications and to assess the return of sensory innervation;
	Preliminary Evaluation of the Clinical Safety and Effectiveness of the Bionic Nerve Scaffold	NCT03780855	Xijing Hospital	The objective of the study is to preliminarily evaluate the clinical safety and effectiveness of the bionic nerve scaffold with longitudinally oriented microchannels;
	Performance Study of an Artificial Nerve Guide (Reaxon® Nerve Guide) to Treat Digital Nerve Lesions	NCT02459015	Medovent GmbH, Several hospitals in Germany	The purpose of this clinical investigation is to confirm the medium- and long-term safety and performance of the chitosan-based nerve guide (Reaxon® Nerve Guide) in comparison to an autologous nerve graft to bridge nerve defects in the finger;
	Chitosan Nerv Tube for Primary Repair of Traumatic Sensory Nerve Lesions of the Hand (CNT)	NCT02372669	BG Unfallklinik, Several hospitals in Germany	The objective of this study is to evaluate whether the additional use of a nerve tube in primary microsurgical repair of traumatic sensory nerve lesions of the hand has an effect on convalescence and functional results;
	Nerve Repair Using Hydrophilic Polymers to Promote Immediate Fusion of Severed Axons and Swift Return of Function	NCT02359825	Vanderbilt University, Vanderbilt University Medical Center	The investigators propose testing the efficacy and safety of a combination therapy: polyethylene glycol (PEG) assisted axonal fusion technique to repair peripheral nerve injuries in humans;
	Do AxoGuard Implants Decrease Shoulder Disability After Neck Dissections?	NCT03941327	University of Mississippi Medical Center	A porcine collagen implant will be used to make a protective sheath around the participant's exposed spinal accessory nerve during surgery. This will be performed by physically wrapping the exposed nerve with the implant and suturing the ends together;
Allografts	Bone Marrow Aspirate Concentrate (BMAC) Nerve Allograft Study	NCT03964129	Brooke Army Medical Center	This study is a prospective, multi-center, proof of principle, phase I human safety study evaluating the sequential treatments of the Avance Nerve Graft, a commercially available decellularized processed peripheral nerve allograft, with autologous Bone Marrow Aspirate Concentrate (BMAC), a source of stem cells;
	A Comparative Post-Marketing Study of Commercially Available Peripheral Nerve Gap Repair Options (CHANGE)	NCT00948025	Axogen Corporation, Several locations across the USA	This study is a comparison of sensory recovery outcomes from the use of AVANCE and hollow tube conduits for peripheral nerve gap repairs in the hand;
	Study of Nerve Reconstruction Using AVANCE in Subjects Who Undergo Robotic Assisted Prostatectomy for Treatment of Prostate Cancer	NCT00953277	Axogen Corporation, Vanderbilt University	The purpose of this study is to determine if it is technically feasible to repair nerves that are injured as part of a planned surgical removal of the prostate and the surrounding tissue in subjects with prostate cancer;
Other grafts	Muscle-in-vein Conduits for Digital Nerve Reconstruction	NCT01958632	BG Trauma Center Tuebingen	The actual study should provide a first direct comparison between results after reconstruction of sensory nerves of the hand using muscle-in-vein conduits to the standard methods of nerve transplantation and direct nerve suture;
Administration of drugs	Safety and Efficacy Study of Neovasculgen (Pl-VEGF165) in Patients with Peripheral Nerve Injury	NCT02352649	Human Stem Cell Institute, Russia	The purpose of this study is to determine safety and efficacy of Neovasculgen for regeneration of peripheral nerve. Neovasculgen is the permitted in Russian Federation angiogenic medication that induce growth of new vessels and included in a complex therapy for patients with peripheral arterial diseases in Russia;
	Tesamorelin to Improve Functional Outcomes After Peripheral Nerve Injury	NCT03150511	Johns Hopkins University	The aim of this clinical trial is to assess the efficacy of tesamorelin as a therapy for peripheral nerve injuries. The investigators hypothesize that treatment with tesamorelin will allow for faster and greater recovery of motor and sensory function following surgical repair of injured peripheral nerves;
	Evaluation of Nicotinamide Riboside in Prevention of Small Fiber Axon Degeneration and Promotion of Nerve Regeneration	NCT03912220	Johns Hopkins University	This study will evaluate the effects of a nutritional supplement called nicotinamide riboside in preventing small fiber nerve degeneration that is experimentally induced by applying capsaicin to skin in otherwise healthy study participants;
	Enhancement of Functional Recovery After Peripheral Nerve Injury with Tacrolimus	NCT00950391	Washington University School of Medicine	The objective of this study is to explore the ability of tacrolimus to benefit the treatment of patients with peripheral nerve injury;
	GW406381 In Patients with Peripheral Nerve Injury	NCT00279032	GlaxoSmithKline, United Kingdom	The findings from preclinical animal models confirm the peripheral anti-inflammatory/analgesic activity of GW406381 and also suggest contribution of a central site of action to the anti-hyperalgesic efficacy that may not be shared by other COX-2 inhibitors;
	Study of Capsaicin Patch for the Management of Peripheral Neuropathic Pain	NCT02228928	Samyang Biopharmaceuticals Corporation	The goal is to test the efficacy and safety of the low concentration [0.65 % (50 μg/cm^2^) and 1.25 % (100 μg/cm^2^)] capsaicin patches and compared them to conventional 0.075 % capsaicin cream and placebo patch in patients suffering from peripheral neuropathy;
	Topical Lidocaine: Predictors of Response in Peripheral Nerve Injury	NCT01112748	Danish Pain Research Center	The primary purpose is to study the predictive value of preserved nociceptors and large afferent fibers and dynamic mechanical allodynia on the effect of lidocaine patch;
	Oxcarbazepine for the Treatment of Chronic Peripheral Neuropathic Pain (IMIOXC)	NCT01302275	Danish Pain Research Center and Innovative Medicines Initiative	The purpose of this trial is to determine if the effect of oxcarbazepine on chronic peripheral nerve pain depends on the supposed mechanism of the pain, ie. if oxcarbazepine mainly relieve pain in patients with irritable nerves;
Cell-based therapies	Emergent Expanded Access for autologous Human Schwann cells Augmentation of Nerve Autografts After Severe Peripheral Nerve Injury	NCT02510079	University of Miami	Schwann cells harvested from the sural nerve will be autologously transplanted along sural nerve autografts wrapped in a collagen matrix (Duragen);
	Safety of Autologous Human Schwann Cells (ahSC) in Subjects with Subacute SCI	NCT01739023	The Miami Project to Cure Paralysis, University of Miami	For humans with subacute SCI, we hypothesize that axons might show improved function if myelin repair is induced with the implantation of ahSC;
	Safety Study of Local Administration of Autologous Bone Marrow Stromal Cells in Chronic Paraplegia (CME-LEM1)	NCT01909154	Puerta de Hierro University Hospital	Follow-up of a cohort of patients with chronic spinal cord injury (SCI) who were treated with autologous stromal cells of the bone marrow administrated locally (subarachnoid and intramedullar) by intrathecal microinjection and 3 months later, by lumbar subarachnoid administration;

The second category analyzed focuses on testing TE NGCs made of different biomaterials and in different sized gaps. Collagen, in the form of a collagen wrap (NCT03875833) or the recently developed Neuramaix (NCT01884376) can be found within this category, as well as a novel silk fibroin NGC (NCT03673449). Furthermore, hydrophilic polymers such as polyethylene glycol have been tested as part of a combination therapy to assist in axonal fusion technique (NCT02359825). In terms of endogenous biomaterials, a clinical trial assessing if AxoGuard implant made of small intestine submucosa ECM decreases disabilities after neck dissections is also currently on going (NCT03941327). A novel synthetic polymer NGC under the name “Polynerve” is also being tested in humans (NCT02970864). Polynerve comprises a co-polymer of PCL and PLA that has the form of a cyçinder containing an internal lumen with detailed micro-grooved architecture.

This last feature also represents an improvement, since NGCs containing any kind of luminal features are rarely found in the phase of clinical trials.

Three clinical trials focusing on the use of allografts have also been found. The first represents a combination of strategies. It conjugates an allograft with a Bone Marrow Aspirate, as source of stem cells, to enhance regeneration (NCT03964129). The second clinical trial is focused on the general use of Avance Nerve Graft, intended to evaluate the general outcomes in a real-life clinical setting (NCT01526681). Using the same allograft, Avance Nerve Graft is being specifically tested in patients who underwent a prostatectomy as the therapy of prostate cancer (NCT00953277). The administration of drugs has been one of the most explored areas in terms of PNR clinical trials. Tesamorelin (NCT03150511), Nicotinamide Riboside (NCT03912220), Tacrolimus (NCT00950391), anti-inflammatory drug GW406381 (NCT00279032), Capsaicin Patch (NCT02228928), topical lidocaine (NCT01112748) and oxacarbazepine for the treatment of chronic pain (NCT01302275) are the adjuvant therapies that have been tested, attempting to reduce some of the symptoms related to PNR.

Regarding cell-therapy, there are numerous pre-clinical reports confirming that cell-based therapy is effective in the treatment of PNI and that it is a promising branch of the PNS regenerative medicine field (Meyer et al., 2016b; Sayad Fathi and Zaminy, 2017). Only one real study carried out in the scope of PNR could be found, in which autologous Schwann cells harvested from the sural nerve are transplanted along sural nerve autografts (NCT02510079). The other cell-based therapy studies that were found relate to the use of cells applied to CNS injuries. Briefly, autologous Schwann cells or autologous Bone Marrow Stromal Cells were injected in subjects with spinal cord injury (NCT01739023) and (NCT01909154), respectively.

Further high-quality research must be done in order to correctly transfer pre-clinical studies into clinical studies in humans, following their translation to the clinics. However, the fact that a variety of strategies mentioned along this review, e.g., luminal guidance structures or cell-based therapies has reached this level in the ladder of clinical acceptance is already thrilling. It means the whole field of PNR is slowly moving to alternatives beyond the hollow NGCs.

## Conclusions

PNIs are permanently accompanied by high costs, translated to economical loss in the health care systems. In addition, there is the morbidity and the significant decrease in patient's quality of life. Therefore, improvements in this field are imperative. There is an increasing agreement that further progress in the field of PNR is urgent, and that the success in the area will no longer be dependent on the new and sophisticated microsurgical tools and techniques or simple hollow NGCs. Instead, due to tremendous quality research recently published, the scientific community is realizing that the best way to move forward is with a multi-combinatorial approach, where different disciplines and specialists may reach a new level of innovation. As such, any approach to peripheral nerve regeneration must integrate a comprehensive and multifaceted strategy, mimicking the natural process of nerve injury and regeneration. *In vivo*, a cascade of events takes place where cellular components, growth factors, topographical and biological cues act together and naturally induce a certain degree of regeneration.

Therefore, developed NGCs, their inherent components and consequent activated mechanisms must enable and simplify the host regenerative process and stimulate regeneration, not only at the injury location, but also at both proximal and distal areas of the injury. Only a multi-factorial device will be capable of overcoming inherent obstacles to regeneration, such as denervation atrophy of muscle targets, lack of vascularization and excessive inflammation.

This report focused on reviewing the diverse approaches that can be used in order to go beyond an empty tubular NGCs, similar to those that have been classically used in the clinics. In one hand, it has been proven that to be successful in treating critical gaps (lengthier than 10 mm, in rat), luminal fillers must be added, to encourage the growth cone in its path-finding quest, to reach the distal target. These luminal fillers comprising 3D structures and fabricated in a variety of ways are absolutely needed in the case of more severe injuries. In this scope, the clinical translation of NeuraGen 3D Nerve Guide Matrix® represented a great step toward the change that needed to be seen in this context. Furthermore, the creation of a favorable milieu in the injury site, created with the exogenous delivery of different biological molecules such as neurotrophic factors, other GFs or the presence of pro-regenerative miRNAs, is fundamental for nerve regeneration. In this area, a wide range of factors and delivery strategies can be engineered, maintaining the principle of a timely fashion release.

Finally, tissue engineers have a panoply of cell-based therapies which can be adopted to amend clinical outcomes. Because of stem cells' potential, they have become a source of cells which act as an alternative to Schwann cells in PNR. Either differentiated or undifferentiated, the mechanisms by which they are valuable in the case of PNI were also explored. All of the above-mentioned features can and should be used to tackle the present hurdles of nerve regeneration. Such tools allow the medical community to perform a more precise work both at the diagnostic as well as in the treatment stages. Additionally, only by surpassing these obstacles and enhancing the velocity and quality of regeneration will allow a proper rehabilitation, where a motor and sensor recovery helps patients regain their quality of life.

Overall, it is crucial to understand that a full functional recovery of PNIs is vital for the patient, to improve its mental health, daily performance, morale, general mobility, agility and capability. All the aforementioned technologies, some of which can already be found in the stage of clinical trials, have the power to go beyond the empty NGCs in terms of positive outcomes. Therefore, they should be adopted and adapted to have a future in the clinical setting, so that patient could benefit from them.

## Author Contributions

CC: conceptualization, investigation, writing—original draft, and visualization. RR: funding acquisition and supervision. JO: writing—review and editing, visualization, supervision, and funding acquisition.

### Conflict of Interest

The authors declare that the research was conducted in the absence of any commercial or financial relationships that could be construed as a potential conflict of interest.
